# High-pressure synthesis and storage of solid organic compounds in active subduction zones

**DOI:** 10.1126/sciadv.abo2397

**Published:** 2022-09-16

**Authors:** Baptiste Debret, Bénédicte Ménez, Bastien Walter, Hélène Bouquerel, Pierre Bouilhol, Nadine Mattielli, Céline Pisapia, Thomas Rigaudier, Helen Myfanwy Williams

**Affiliations:** ^1^ Université Paris Cité, Institut de physique du globe de Paris, CNRS, Paris, France.; ^2^ Université de Lorraine, CNRS, GeoRessources, Vandoeuvre-lès-Nancy, France.; ^3^ Université de Lorraine, CNRS, CRPG, Nancy, France.; ^4^ Laboratoire G-Time, DGES, Université Libre de Bruxelles (ULB), Brussels, Belgium.; ^5^ Department of Earth Sciences, University of Cambridge, Cambridge, UK.

## Abstract

Recent thermodynamic and experimental studies have suggested that volatile organic compounds (e.g., methane, formate, and acetate) can be produced and stabilized in subduction zones, potentially playing an important role in the deep carbon cycle. However, field evidence for the high-pressure production and storage of solid organic compounds is missing. Here, we examine forearc serpentinite clasts recovered by drilling mud volcanoes above the Mariana subduction zone. Notable correlations between carbon and iron stable-isotope signatures and fluid-mobile element (B, As and Sb) concentrations provide evidence for the percolation of slab-derived CO_2_-rich aqueous fluids through the forearc mantle. The presence of carbonaceous matter rich in aliphatic moieties within high-temperature clasts (>350°C) demonstrates that molecular hydrogen production associated with forearc serpentinization is an efficient mechanism for the reduction and conversion of slab-derived CO_2_-rich fluids into solid organic compounds. These findings emphasize the need to consider the forearc mantle as an important reservoir of organic carbon on Earth.

## Teaser

The study of forearc ultramafic clasts provides evidence for a deep organic carbon cycle in subduction zones.

## INTRODUCTION

In modern Earth, the extraction of carbon from the mantle to the surface plays a major role regulating the temperature, composition, and oxygenation levels of the atmosphere and hydrosphere (*1*–*3*). Mantle carbon extraction mainly occurs at mid-ocean ridges and subduction zones through mantle partial melting and magmatic degassing processes (*4*, *5*). Carbon is then returned to the mantle via subduction zones, although the nature and the fraction of carbon that is recycled into Earth’s interior remains controversial (*6*–*8*). The sedimentary, crustal, and mantle parts of the subducting slab and the mantle wedge are all potential hosts for inorganic and organic carbon in subduction zones. However, while many studies have provided estimates of the budgets and distribution of inorganic and organic carbon in the oceanic crust and its sedimentary cover (*9*–*13*), relatively little is known about the role that mantle rocks may play in the subduction zone carbon cycle, especially regarding organic carbon.

At Earth’s surface, the hydration of mantle-derived ultramafic rocks (a series of complex reactions encompassed under the term serpentinization) plays a major role in the conversion of inorganic carbon, such as CO_2_ or CO, into organic compounds, made of carbon and hydrogen often associated with heteroatoms, such as oxygen or nitrogen, via abiotic redox reactions (*14*). In these reactions, molecular hydrogen (H_2_) is produced from water reduction predominantly associated with the oxidation of primary Fe^2+^-bearing minerals to form Fe^3+^-bearing minerals, such as magnetite at high temperatures (>200°C) or serpentine at low temperatures (<200°C) (*15*, *16*). The reducing power of H_2_ is widely recognized as a source for the abiotic production of hydrocarbons (C*_n_*H*_m_*), including methane (CH_4_) and volatile organic acids (e.g., formic acid) detected in hydrothermal fluids [e.g., (*17*)].

Recent advances in in situ spectroscopic techniques have allowed the identification of serpentinization-derived solid organic compounds in abyssal serpentinites (*18*–*20*). These results provide fundamental evidence for the abiotic synthesis and storage of a diverse range of organic compounds in these rocks, including carbonaceous matter related to polycyclic aromatic hydrocarbons (PAHs). It is now recognized that there is a broad range of environments across Earth’s surface and interior that may support conditions suitable for the abiotic synthesis of both solid and volatile organic compounds and that these processes are not limited to low-pressure (LP) hydrothermal environments. In particular, solid carbon compounds represent a host for carbon in metamorphic rocks and may represent an important, although largely unconstrained, fraction of carbon in the lithosphere, potentially contributing to the deep carbon cycle on Earth. Recent thermodynamic (*21*) and experimental (*22*, *23*) studies show that the formation and stability of organic compounds in metamorphic fluids are predominantly controlled by variations in oxygen fugacity (*f*O_2_) and pH, as opposed to pressure and temperature. Abiotic redox reactions leading to the synthesis of diverse organic components may therefore occur at depth in subduction zones, despite the extreme temperatures and pressures.

Although organic compounds (e.g., CH_4_, formate, and acetate) are predicted to be present as dissolved species in slab-derived fluids (*21*), no consensus for the formation, role, and variability of solid organic compounds in subduction zones exists. At this time, few natural occurrences of organic compound formation in high-pressure (HP) metamorphic fluids have been documented, although exclusively limited to orogenic settings. For example, the observation of H_2_- and/or CH_4_-rich fluid inclusions in HP ultramafic rocks from the Western Alps (France, Italy) and Vermont (USA) mountain belts provides pieces of evidence for the circulation of reduced and organic-rich fluids in paleosubduction zones (*24*–*26*). Vitale-Brovarone *et al.* (*24*) proposed that the production of H_2_-rich fluids during mantle peridotite serpentinization at HP and their interaction with carbonate minerals could generate abiotic CH_4_. Under favorable conditions, these H_2_-CH_4_ fluids may also favor the abiotic precipitation of abiotic graphite-bearing lithologies as they migrate through the mantle (*27*, *28*). In addition, Bouilhol *et al.* (*29*) observed solid organic compounds with variable relative content in aliphatic and aromatic moieties in HP-ultramafic rocks from eclogite-facies Alpine meta-ophiolites (550° to 600°C, 2 to 2.5 GPa), which demonstrates that organic compounds are not limited to fluids but can also exist and persist as rock-hosted organic matter in HP environments. These observations are consistent with thermodynamic (*30*) and experimental (*31*) studies that have shown a progressive increase in PAH stability in rocks relative to fluids with increasing pressure. Furthermore, at HP, the formation of PAHs requires lower activity of carbon and H_2_ relative to LP environments (*30*). It is therefore conceivable that abiotic synthesis of solid organic compounds could be highly efficient in extreme environments, such as subduction zones, favoring the storage of organic carbon in rocks relative to fluids and potentially providing an important global reservoir for carbon. Critically, the formation and stabilization of these solid organic compounds will exert a major control on subduction zone redox reactions and *f*O_2_, as for example, the conversion of carboxylic acids into methane requires an addition of C―H or H_2_ (hydrogenation) and/or a loss of oxygen through redox processes. However, although solid organic compounds have been observed in active subduction settings, their presence has been ascribed to microbial activity associated with LP retrograde processes rather than abiotic reactions (*32*). Furthermore, there are almost no constraints on the nature and abundance of solid organic compounds generated at HP through abiotic redox processes in subduction zones and their potential role in the deep carbon cycle. Here, we address this knowledge gap and revisit the abiotic and biological formation of solid organic compounds in the active Mariana subduction system through a study of serpentinized clasts recovered during the Integrated Ocean Discovery Program (IODP) Expedition 366 from mud volcanoes formed above the Mariana forearc. We demonstrate that the abiotic synthesis of solid organic compounds in the forearc mantle wedge results from the reduction of CO_2_-rich slab-derived fluids at HP and high temperatures (>350°C). We show that although solid inorganic carbonates are abundant near the surface, they relate to shallow microbial activity while, with increasing depth, rock-hosted solid organic compounds become the main carrier of carbon, calling for a reappraisal of serpentinized forearc as a major reservoir for abiotic production and storage of organic carbon in subduction systems.

## RESULTS

### Geological setting and sample selection

**Fig. 1.**
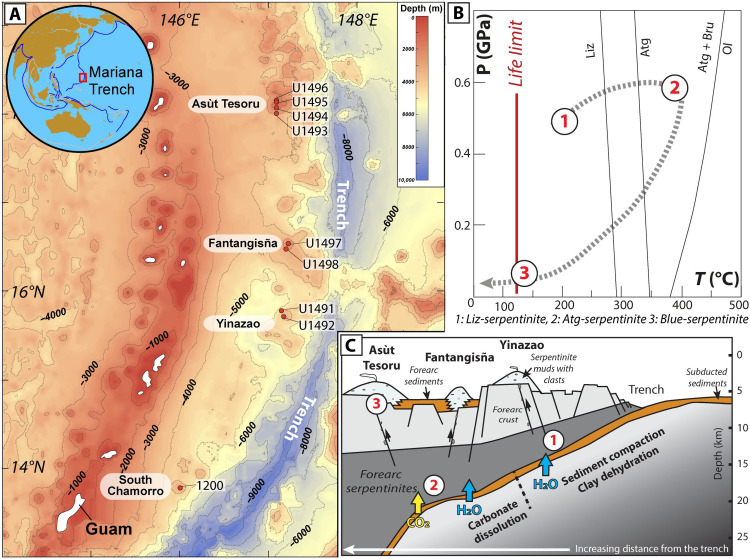
Bathymetry map, pressure and temperature (*P*-*T*) path and conceptual cross section of the Mariana forearc system. Figure modified after (*36*, *37*). (**A**) The map shows the locations of the three mud volcanoes, Yinazao, Fantangisña, and Asùt Tesoru, drilled during IODP Expedition 366. Hole locations are indicated in red circles. (**B**) *P*-*T* path presenting the different conditions of serpentinization recorded by the sampled forearc ultramafic clasts (i.e., Liz-, Atg-, and blue-serpentinites, from 1 to 3, respectively). Life limit is after Kashefi and Lovley (*46*). (**C**) Conceptual model illustrating serpentinization processes in relation to fluid circulation and mantle flow within the Mariana forearc.

The Mariana subduction zone belongs to a nonaccretionary subduction system, spreading from near Tokyo (Japan) to beyond Guam (USA) and involving the subduction of the Mesozoic Pacific plate below the Philippine Sea plate (Fig. 1A). It is the only place in the world where active metasomatized forearc can be sampled (*33*–*35*). Along the Mariana forearc, serpentinite mud volcanoes erupt as a consequence of the interaction of aqueous fluids released from the dehydrating subducting slab with forearc mantle wedge peridotites. These muds bring to the seafloor a cargo of serpentinized forearc–derived peridotite clasts that provide a direct window on chemical exchange between the slab and the forearc mantle at various depths. The clast samples studied here were recovered by drilling three mud volcanoes, namely, Yinazao, Fantangisña, and Asùt Tesoru, located at increasing distance from the trench (Fig. 1A). Three types of serpentinized forearc peridotite clasts, namely, lizardite, antigorite, and blue serpentine–bearing clasts, were identified on the basis of their petrographic and geochemical features (*35*–*37*). These clasts record various stages of serpentinization from deep forearc mantle hydration by slab-derived fluids to shallow fluid-rock interaction during clast exhumation nearby the seafloor (Fig. 1, B and C).

The lizardite- and antigorite-bearing serpentinite clasts preserved a record of deep forearc mantle serpentinization (Fig. 1, B and C) by slab-derived fluids and do not display evidence for notable retrogression (e.g., blue-serpentine or sulfide crystallization, late-stage carbonation) (*36*). The lizardite-bearing serpentinites (Liz-serpentinites) display variable degrees of serpentinization (30 to 100%, as defined by petrographic observations and bulk rock geochemical analyses) and are primarily composed of low-temperature brown serpentine (i.e., lizardite and/or chrysotile) and Fe-rich brucite assemblages forming mesh and bastite textures after olivine and orthopyroxene, respectively (fig. S1A). These rocks represent the first stage of forearc mantle wedge hydration at low temperatures (180° to 230°C, based on oxygen isotope estimates) and shallow depths (<13 km) (Fig. 1B) (*36*). The antigorite-bearing serpentinites (Atg/Liz- and Atg-serpentinites) are highly serpentinized (~100%) and display progressive replacement of lizardite-bearing textures (i.e., mesh and bastite) by antigorite, magnetite, and Fe-poor brucite ± andradite (fig. S1B). Oxygen isotope thermometry data suggest that the recrystallization of lizardite into antigorite occurred at temperatures ranging from 250° to 320°C (*36*), consistent with thermodynamic predictions (*38*) and natural temperature estimates (*39*) for antigorite crystallization in subduction zones (Fig. 1B). These observations provide evidence for the formation of these samples during the progressive burial and hydration of forearc mantle at depths ranging from 13 to 18 km (*36*).

The blue-serpentinites are considered to be former forearc peridotites or serpentinites (i.e., Liz- or Atg-serpentinites) recrystallized at low temperatures (<130°C) during the final stages of clast exhumation. These samples are highly serpentinized (serpentinization degree >80%) and are characterized by the crystallization of serpentine textures (i.e., mesh or bastite textures) with a strong blue color (fig. S1C). These blue-serpentine textures largely overprint any primary mantle mineral or former serpentine (i.e., lizardite and antigorite) textures. These textures can also be associated with framboidal sulfides (mainly pyrite), probably formed by tardive bacterially mediated sulfate reduction (*36*) and Fe hydroxides.

### Iron distribution in forearc ultramafic clasts

The Liz-serpentinite clasts display high Fe^3+^/ΣFe [= Fe^3+^/(Fe^2+^ + Fe^3+^)] ratios, ranging from 0.24 to 0.37 (table S1), relative to the primitive mantle (Fe^3+^/ΣFe ~ 0.1) (*40*). In these clasts, opaque phases form large subautomorphic crystals that are interpreted as relict mantle spinels (fig. S1A). Magnetic susceptibility and Curie temperature measurements (figs. S2 and S3) reveal that magnetite is nearly absent, with modal amount estimates ranging from <0.1 to 0.7 weight % (wt %; table S2). In agreement with these observations, only rare and small (<50-μm width) magnetite-bearing veins were observed by x-ray computed tomography (CT) analyses and in thin section (fig. S1A). The near-total absence of magnetite despite high bulk rock Fe^3+^/ΣFe suggests the preferential partitioning of Fe^3+^ into serpentine minerals, in agreement with the inferred low temperatures of serpentinization (<200°C) (*15*). The Atg/Liz- and Atg-serpentinites display even higher Fe^3+^/ΣFe ratios (0.33 to 0.67; table S1) relative to Liz-serpentinites. In these clasts, the growth of antigorite is associated with the formation of a dense network of discrete and elongated particles, identified as magnetite, organized along a single preferred orientation (fig. S1B). The magnetic susceptibility and Curie temperature measurements (figs. S2 to S3) confirm that magnetite is the main magnetic phase crystallizing in Atg-serpentinites, with modal amount estimates ranging from 0.2 to 5.9 wt % (table S2). Magnetite crystallization is always associated with Fe-poor brucite and sometimes, to a lesser extent, andradite (*36*).

The blue-serpentinites are characterized by a large range in Fe^3+^/ΣFe (0.33 to 0.77; table S1). Curiously, samples with high Fe^3+^/ΣFe ratios display relatively low magnetite contents (<1 wt %; fig. S2 and table S2), and Curie temperature measurements reveal an irreversible behavior during successive heating and cooling (fig. S3). These features can be attributed to the presence of sulfate and/or Fe sulfides (e.g., greigite), which are weakly magnetic and are irreversibly converted to magnetite and/or pyrrhotite during Curie temperature measurements. This is supported by x-ray CT analyses that show the existence of isolated large pyramidal sulfides in those rocks (fig. S1C). The quasi-absence of magnetite in blue-serpentinites and the irreversible magnetic behavior observed during Curie temperature measurements suggest that serpentine is the main Fe^3+^ carrier in those clasts. These observations are consistent with low temperatures of serpentinization favoring the incorporation of Fe^3+^ in serpentine minerals (*15*) coupled with oxidizing conditions accompanying near-seafloor alteration (*35*).

### Carbon bulk rock isotope analyses

Seventeen representative samples were analyzed for bulk carbon elemental and isotope compositions. Carbon analyses were performed for both inorganic [TIC (total inorganic carbon)] and organic [TOC (total organic carbon)] carbon compounds in addition to total carbon (TC; table S3). The Mariana forearc serpentinite clasts have TC concentrations [(C_TC_) = 389 to 12,523 parts per million (ppm)] and isotope compositions (δ^13^C_TC_ = −11.8 to −25.5‰) that overlap with those of abyssal and HP-serpentinites (fig. S4A). There are no systematic variations in (C_TC_) or δ^13^C_TC_ from Liz- (408 to 637 ppm; −11.8 to −19.2‰) to Atg/Liz- (403 to 1960 ppm; −12.4 to −17.7‰), Atg- (389 to 1142 ppm; −14.0 to −21.3‰), or blue-serpentinites (652 to 12,523 ppm; −15.6 to −25.5‰), the latter group displaying the most extreme variations in both (C_TC_) and δ^13^C_TC_.

To put these results in context, the TC concentrations (C_TC_) of the Mariana forearc serpentinite clasts are significantly greater than that inferred for the depleted mantle [(C_TC_) = 50.3 ppm] (*41*). Moreover, the Mariana forearc serpentinites are characterized by similar total concentration of organic carbon (C_TOC_) (194 to 10,291 ppm) and δ^13^C_TOC_ (−23.3 to −29.4‰) relative to those of abyssal (*42*, *43*) or HP-serpentinites (*44*, *45*) (fig. S4B). However, these samples also display highly variable concentration of TIC (C_TIC_) (67 to 2323 ppm) and δ^13^C_TIC_ (−2.2 to −16.3‰), with a few samples having abnormally low δ^13^C_TIC_ when compared with literature data for similar samples (fig. S4C).

### Carbon microimaging

**Fig. 2.**
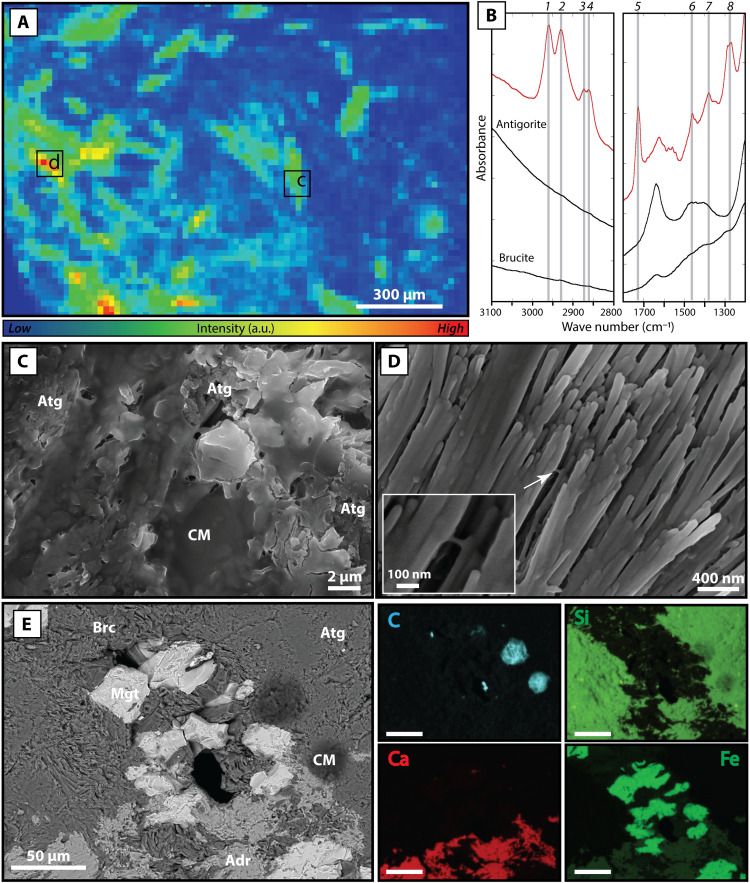
Characterization of carbonaceous matter wetting antigorite-bearing Mariana forearc serpentinites. (**A**) FTIR map of the aliphatic CH_2_-CH_3_ C─H stretching band area between 2800 and 3000 cm^−1^ showing the distribution of organic carbon encountered in an Atg-serpentinite. Areas depleted in organic compounds (blue) spatially correlate with the presence of Fe-poor brucite ±antigorite, whereas the organic signal (from green to red) is systematically associated with antigorite. The organic fraction appears chemically homogeneous throughout the analyzed areas (fig. S6). a.u., arbitrary unit. (**B**) Examples of raw FTIR spectra of brucite, antigorite, and carbonaceous matter associated with antigorite (in red), extracted from the map shown in (A). The organic-related spectrum is characterized by bands at 1: ~2960 cm^−1^, CH_3_ asymmetric C─H stretching, 2: ~2920 cm^−1^, CH_2_ asymmetric C─H stretching, 3: ~2870 cm^−1^, CH_3_ symmetric C─H stretching, 4: ~2850 cm^−1^, CH_2_ symmetric C─–H stretching, 5: ~1730 cm^−1^, C═O stretching of aliphatic aldehyde, 6: ~1470 cm^−1^, CH_3_ asymmetric C─H bending, CH_2_ scissoring, and 7: ~1380 cm^−1^, CH_3_ symmetric C─H bending. Band assignment are from (*71*, *72*). (**C** and **D**) SEM-SE (secondary electron) images of carbonaceous matter (CM) films embedding antigorite. (C) The discontinuous carbonaceous matter film covers the mineral surface outcropping at some places. (D) The film of carbonaceous matter completely embeds antigorite needles, locally connecting two crystals (white arrow with magnified view as inset). (**E**) SEM-BSE (backscatter electron) image with associated EDS element maps (C, Si, Ca, and Fe) of an Atg-serpentinite showing the relationship between CM and the HP paragenesis, made of antigorite, Fe-poor brucite (Brc), andradite (Adr), and magnetite (Mgt).

**Fig. 3.**
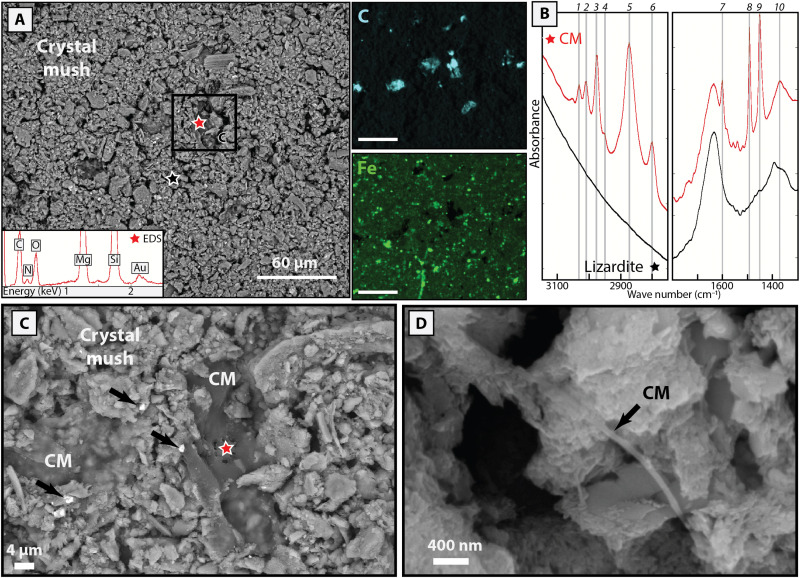
Carbonaceous matter characterization in blue-serpentinites. (**A**) SEM-BSE image and associated EDS element maps for carbon and iron in a mesh core crystallizing in blue-serpentinites. The mesh core is composed of a crystal mush with a high porosity and containing brucite, lizardite, and Fe hydroxides and sulfides. The mush porosity is filled with carbonaceous matter. The red and black stars correspond to the localization of punctual EDS (shown in inset) and FTIR (B) analyses. The black inset locates (C). (**B**) Raw FTIR spectra of the carbonaceous matter (in red) associated with lizardite (in black). The organic-related spectrum is characterized by bands at 1 to 4: 3105, 3082, 3061, and 3028 cm^−1^, aromatic C─H stretching, 5 and 6: ~2920 and ~ 2850 cm^−1^, CH_2_ asymmetric and symetric C─H stretching, 7 to 9: 1603, 1493, and 1450 cm^−1^, C═C stretching ± CH_2_ scissoring, 1493 cm^−1^, and 10: 1370 cm^−1^ aromatic C─N stretching. (**C**) Magnified SEM-SE image of carbonaceous matter filling the interstitial porosity of the crystal mush. Black arrows indicate micro–Fe hydroxides and sulfides associated with the crystal mush. (**D**) SEM-SE image of carbonaceous matter with a tubular texture.

The serpentinite clasts contain significant and highly variable amounts of carbon (bulk-rock concentrations of 400 ppm to 1.2 wt %; table S3). Although no carbon-bearing minerals (e.g., calcite) and graphite are observed in thin sections, Fourier transform infrared (FTIR) microimaging shows the presence of large areas (several hundreds of micrometers wide) of carbonaceous matter displaying vibrational bands characteristic of organic compounds in the Atg- and blue-serpentinites (Figs. 2 and 3).

In the Atg-serpentinites, the FTIR spectra of the carbonaceous matter are characterized by intense methyl (CH_3_─) and methylene (CH_2_─) C─H stretching bands within the 3000 to 2800 cm^−1^ and 1500 to 1200 cm^−1^ ranges, corresponding to aliphatic chains and by a marked band characteristic of oxygen-bearing functional groups at ~1730 cm^−1^ (C═O from aliphatic aldehyde) (Fig. 2B). The computed *R*_CH2/CH3_ ratio does not exceed 1, giving a raw carbon number for the aliphatic chains below 8 to 10 when isomers are considered. The organic signal in the FTIR spectra is consistently associated with additional broad bands around 1640, 1460, and 1400 cm^−1^, which correspond to antigorite. Scanning electron microscopy (SEM) observations reveal that carbonaceous matter forms large patches up to 100 μm wide, either covering the surface of antigorite crystals (Fig. 2, C to E) or forming thin films embedding antigorite needles (Fig. 2D). These films have a gel-like appearance and occasionally connect bathed antigorite crystals (Fig. 2E). Carbonaceous matter was also observed at the interface between antigorite and brucite or andradite crystals (Fig. 2E), although this was rare.

In blue-serpentinites, the carbonaceous matter show a contrasted FTIR spectral signature compared to Atg-serpentinites, with methyl and methylene C─H stretching bands at 2850 and 2925 cm^−1^, corresponding to aliphatic chains, associated with aromatic C─H stretching bands at 3025, 3060, and 3085 cm^−1^ (Fig. 3B). Three additional marked bands at 1603, 1493, and 1450 cm^−1^ can be assigned to aromatic C═C stretching (± CH_2_ scissoring) and an additional band at 1370 cm^−1^ can be attributed to C─N stretching. SEM observations show that the carbonaceous matter is located in a porous crystal mush of serpentine, brucite, andradite, and Fe-rich phases (including Fe sulfides and Fe hydroxides; Fig. 3A). The carbonaceous matter precipitates as gelatinous patches of about 50 μm in diameter, filling the textural porosity (Fig. 3, A to C). Magnified images reveal that the organic matter can also display a tubular filament-like texture (Fig. 3D). Energy dispersive x-ray spectrometry (EDS) analyses show that this carbonaceous matter signal is associated with a marked nitrogen peak (Fig. 3A), suggesting that the solid organic compounds observed in the blue-serpentinites may potentially host nitrogen.

## DISCUSSION

### Deciphering the biologic versus abiotic origin of HP carbonaceous matter

**Fig. 4.**
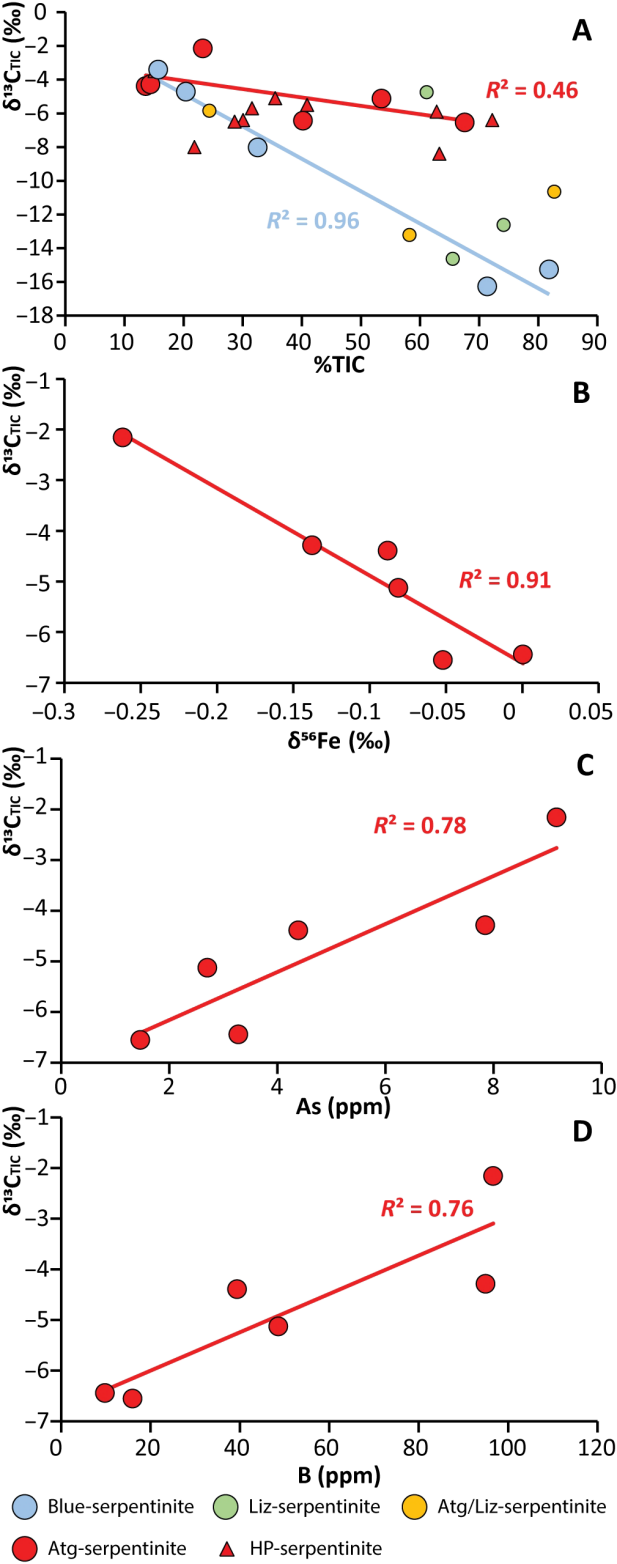
Covariations of carbonate isotopic composition in serpentinite clasts with elemental tracers. (**A**) δ^13^C_TIC_ versus carbonate abundance (%TIC). (**B**) δ^13^C_TIC_ versus δ^56^Fe. (**C**) δ^13^C_TIC_ versus As. (**D**) δ^13^C_TIC_ versus (B). Plots (B to D) only show data for Atg-serpentinites. HP-serpentinite data are from (*44*, *45*).

The crystallization of the blue-serpentinites is considered to have taken place during clast exhumation, in subsurface low-temperature environments (Fig. 1), as shown by δ^18^O temperature estimates (<180°C) (*36*) and the low magnetite modal amounts (figs. S2 and S3 and table S2) determined for these samples. These conditions are compatible with microbial activity (i.e., ≤121°C, the current established limit for life) (*46*) and are comparable to those estimated by Plümper *et al.* (*32*), who consider subduction zones as the largest terrestrial microbial habitat, using the example of the Mariana forearc system. The δ^13^C_TIC_ compositions of the blue-serpentinites are highly heterogeneous, varying from −3.4 to −16.3‰. These values are abnormally “light” (depleted in heavy carbon isotopes) relative to the δ^13^C_TIC_ compositions of abyssal (from 6.2 to −9.9‰) or HP-serpentinites (from 1.4 to −8.4‰) (fig. S4C) (*46*). These δ^13^C_TIC_ variations are correlated with a decrease in TIC (%TIC) in the bulk rock, with organic-rich samples displaying high δ^13^C_TIC_ signatures and inorganic carbon–rich samples having low δ^13^C_TIC_ signatures (Fig. 4A). This observation is unexpected as carbonates are known to preferentially uptake isotopically “heavy” carbon (enriched in heavy carbon isotopes), while organic carbon is expected to preferentially uptake isotopically light carbon (*42*, *43*). Such low δ^13^C_TIC_ values may therefore reflect the oxidation of isotopically light organic carbon into carbonate, such that the carbonate inherits an “organic carbon” isotope signature, e.g.

$${\text{CH}}_{4}+2H_{2}O={\text{CO}}_{2}+4H_{2}$$ (1)

This mechanism is commonly observed in passive margins where authigenic carbonates displaying abnormally low δ^13^C_TIC_, down to −60‰, are formed by anaerobic microbial consortia through syntrophic methane (± other hydrocarbons) oxidation and sulfate reduction (*47*, *48*)

$${\text{CH}}_{4}+{{\text{SO}}_{4}}^{2‐}={{\text{HCO}}_{3}}^{‐}+{\text{HS}}^{‐}+H_{2}O$$ (2)

The microbial oxidation of organic carbon compounds in blue-serpentinites is also supported by the predominance of authigenic aragonite and calcite in the top meters of the mud volcanoes (*49*–*51*). On the basis of Sr and C isotope analyses, their precipitation was interpreted as resulting from microbially mediated fluid interactions between shallow seawater and deep-sourced and slab-related fluids. Furthermore, in the blue-serpentinites, carbonaceous matter is found within serpentinite pore spaces, in crystal mushes composed of lizardite, brucite, andradite, Fe sulfide, and Fe hydroxides (Fig. 3, A to D). This close association of carbonaceous matter with sulfides, which can display framboidal textures (*36*), is compatible with Eq. 2, the later textures being commonly attributed to sulfate-reducing bacteria (*52*). Microbial activity is also supported by the tubular, i.e., filament-like, textures sometimes found in blue-serpentinites (Fig. 3D) and the spectral signature of the carbonaceous matter, which is composed of a mixture of nitrogen-bearing aliphatic and aromatic organic compounds (Fig. 3B), as described by Plümper *et al.* (*32*). Together, these geochemical and petrographic observations support the biologically mediated breakdown of organic compounds to form carbonate minerals during exhumation of the blue-serpentinites to near-seafloor depths.

Compared to the blue-serpentinites, the Atg-serpentinites display relatively homogeneous δ^13^C_TIC_ values (−2.2 to −6.6‰) for a large range of %TIC (13.6 to 67.6%). No obvious correlation between δ^13^C_TIC_ and %TIC is observed (Fig. 4A). The δ^13^C_TIC_ values of these samples also fall within the range traditionally observed for HP-serpentinites (Fig. 4A and fig. S4C), as do the carbon isotopes values of TOC and TC. Cannao *et al.* (*45*) postulate that the devolatilization of carbon-rich sediments under oxidizing conditions during subduction is likely to produce fluids with relatively low δ^13^C_TIC_ (<0‰) that could then react with peridotites and/or HP-serpentinites and affect low δ^13^C_TIC_ signatures to these rocks. The similar δ^13^C_TIC_ signatures of HP-serpentinites and the Atg-serpentinites suggest that fluids derived from the breakdown of carbon-rich slab sediments under oxidizing conditions may also be important in forearc peridotite serpentinization. Strong correlations between bulk rock concentrations of fluid mobile elements (FMEs) such as As and B, which are characteristic of sedimentary components in subduction zones (*53*–*55*), and δ^13^C_TIC_ in the Atg-serpentinites (Fig. 4, C and D) further support the mobilization of carbonate derived from fluids released by sediment during subduction.

In the Atg-serpentinites, carbonaceous matter forms thin gel textures covering antigorite or patches filling the interstices between brucite, magnetite, and andradite. The close and systematic association of gel-like carbonaceous matter wetting antigorite suggests that these phases are syngenetic. In addition, the high crystallization temperatures of antigorite in the forearc (310° to 410°C; Fig. 1B) (*36*) relative to those considered compatible with life (≤121°C) (*46*), the textural association between carbonaceous matter and antigorite provides additional evidence for an abiotic origin of the carbonaceous matter. Consistent with this interpretation, the FTIR spectral signature of the Atg-serpentinite carbonaceous matter differs to those observed in blue-serpentinites. In these samples, the FTIR spectral signature is characterized by a strong aliphatic organic signal, with carbon number <8 to 10, high content in O-bearing functional groups, and a low level of aromatic moieties (Fig. 2), suggestive of organic acids that are common by products of abiotic organic synthesis.

Overall, these observations suggest that biotic and abiotic processes lead to the formation of solid organic compounds with different petrological habits, as well as geochemical and FTIR spectral signatures in blue- and Atg-serpentinites. The Liz- and Atg/Liz-serpentinites display intermediate δ^13^C_TIC_ (−4.7 to −14.6‰) and %TIC (24 to 83%) between blue- and Atg-serpentinites when plotted on Fig. 4A. Notably, these samples do not display any correlations between δ^13^C_TIC_ and FME concentrations, suggesting that these samples and the blue-serpentinites do not record fluids released by sediment during subduction. This observation combined with low temperature of serpentinization (*36*) suggest that these samples were serpentinized at shallow depths by CO_2_-free fluids.

### Linking redox reactions to HP abiotic synthesis and storage of solid carbon compounds

In the deep part of the forearc (i.e., > 10-km depth), the serpentinization of mantle peridotites through interaction with water-rich fluids derived from the slab is commonly associated with the oxidation of ferrous Fe leading to a progressive increase of Fe^3+^/ΣFe in the bulk rock and H_2_ production in the fluid phase through water reduction

$$3\;\text{FeO}+H_{2}O_{\left(\text{aq}\right)}={\text{Fe}}_{3}O_{4}+H_{2(\text{aq})}$$ (3)

In agreement with this, the Mariana serpentinized clasts display high Fe^3+^/ΣFe ratios (0.24 to 0.67) relative to mantle peridotites (~0.1) (*40*). However, for a given serpentinization degree, the Atg-serpentinites display higher Fe^3+^/ΣFe ratios than Liz- or Atg/Liz-serpentinites (fig. S5), which is consistent with the progressive recrystallization of serpentinite at high temperatures and an increase in the modal amount of magnetite in bulk rock (fig. S2). These results demonstrate the progressive oxidation of iron through the high-temperature recrystallization of lizardite and Fe-rich brucite into antigorite, Fe-poor brucite, and magnetite. Negative correlations have also been observed between Fe^3+^/ΣFe and serpentinite bulk rock stable iron isotopes (δ^56^Fe) in the same sample suite (*37*). Debret *et al.* (*37*) interpreted these correlations and the high Fe^3+^/ΣFe ratios of Atg-serpentinites in terms of the transfer of slab-derived CO_2_-bearing fluids to the forearc mantle wedge. In this scenario, these fluids preferentially complex isotopically light Fe and can modify the redox state of the forearc mantle at high temperature according to the simplified reaction

$${\text{FeCO}}_{3(\text{aq})}+3\text{FeO}=2\;{\text{Fe}}_{2}O_{3}+C$$ (4)

A notable correlation between δ^13^C_TIC_ values, which are believed to retain the isotopic signature of sediment-derived fluids, and δ^56^Fe values is observed in the Atg-serpentinites (Fig. 4B). Such a correlation is not observed in blue-serpentinites, where the carbon isotope signature appears to have been reset by microbial activity associated with clast exhumation. Any late carbon recrystallization in an open system or redistribution in a closed system during clast exhumation should induce an isotope fractionation of δ^13^C_TIC_ and would destroy any correlations between δ^13^C_TIC_ and δ^56^Fe, as for example, seafloor-related serpentinization is not associated with Fe isotope fractionation (*56*). We therefore interpret the correlation between δ^13^C_TIC_ and δ^56^Fe in the Atg-serpentinites in terms of transfer of oxidizing fluids (e.g., CO_2_) from slab to the forearc mantle at depth. Such a scenario is consistent with the large fluxes of subducting carbonate-rich sediments reported at ODP legs 129 and 189 nearby Mariana trench that carry an isotopically heavy signature in carbon (in average, 2.3‰) (*57*). Heavy carbon isotope signatures (−0.5‰) have also been reported by Hilton *et al.* (*58*) in geothermal fluids (from geothermal wells, fumaroles, and hot springs) collected from the Mariana Arc islands. These authors conclude that 87% of the emitted CO_2_ in Mariana volcanic system is slab derived. Similarly, Resing *et al.* (*59*) analyzed He isotopes in hydrothermal fluids from active submarine volcanoes and proposed that >80% of the CO_2_ venting from these volcanoes had a slab source. Further evidence in favor of a slab origin comes in the form of combined ^143^Nd/^144^Nd and ^138^Ce/^142^Ce isotope data for Mariana volcanic rocks, which reveals a strong subducted sedimentary component in the source of Mariana arc system (*60*). In contrast, the He and C isotope signatures of Mariana back arc basalts are indistinguishable from those of mid-ocean ridge basalt, suggesting a mantle source–like signature (between −6.1 and −5.5‰) and, hence, limited carbon transfer beyond subarc depth (*61*). Overall, these observations suggest that most of carbon transfer between the slab and the mantle wedge occurs under oxidized form (e.g., CO_2_) during the first stages of subduction, placing the serpentinized forearc mantle in an ideal position to become a carbon sink in this system.

Despite recording the transfer of CO_2_ in slab-derived fluids, the carbon budget of the Atg-serpentinites is, in fact, dominated by solid organic carbon species presumably formed by the reduction of the fluid-derived CO_2_ component. The exact forms of organic carbon that can be formed through these redox processes (e.g., Eq. 4) operating in the forearc are numerous and depend on carbon sources, redox conditions associated with slab decarbonation, and local chemical and redox equilibria. For example, oxidized conditions may lead to a wide variety of organic compounds in addition to inorganic carbon species, as suggested by the O-bearing functional groups associated with the carbonaceous matter in Atg-serpentinite (Fig. 2B). The transfer of both H_2_O (Eq. 3) and CO_3_^2−^ (Eq. 4) to the forearc mantle may therefore also lead to the abiotic synthesis of organic acids, e.g.

$$2\;{\text{FeCO}}_{3(\text{aq})}+6\;\text{FeO}+2\;H_{2}O_{\left(\text{aq}\right)}=4\;{\text{Fe}}_{2}O_{3}+{\text{CH}}_{3}{\text{COO}}^{‐}+{H^{+}}_{\left(\text{aq}\right)}$$ (5)

In agreement with such a scenario, high concentrations of dissolved H_2_ (10 to 1000 μM), CH_4_ (1 to 10,000 μM), and C_2_H_6_ (0.1 to 1000 μM), as well as formate (HCOO^−^, ~100 μM) and acetate (CH_3_COO^−^, ~40 μM) organic acids were measured in the interstitial waters most distant to the trench mud volcanoes (i.e., Asùt Tesoru, South Chamorro, and Conical), which are considered to sample the deepest fluid/rock interactions between the slab and the forearc mantle wedge (*35*, *62*, *63*). In contrast, comparatively low levels (H_2_ = 1 to 100 μM; CH_4_ = 0.1 to 10 μM; C_2_H_6_ = not determined, HCOO^−^ ~2 μM; CH_3_COO^−^ ~ 2 μM) were detected at the mud volcanoes located close to the trench (i.e., Yinazao) reflecting the limitation of carbonate reduction processes in the shallow parts of the forearc (*35*, *62*). The link between serpentinization reactions and organic carbon production with depth is also supported by the presence of solid organic compounds associated with antigorite and that have a strong aliphatic signature (Fig. 2). It shows that the rate of iron oxidation and, consequently, the H_2_ production increases with depth where it enhances the reduction of CO_2_-bearing slab fluids. This results in the formation of organic carbon compounds in the form of organic carbon-rich fluids discharged through mud volcanoes or carbonaceous matter with an aliphatic signature trapped within mantle rocks. Given that the solubility of alkanes decreases with carbon number (*64*) and that organic acids >C5 are insoluble in water owing to hydrophobicity of long aliphatic chains, we hypothesize that both the volatile hydrocarbons and organic acids detected at mud volcanoes and the clast-hosted carbonaceous matter may derive from the same redox reactions, with the fraction soluble in water, feeding hydrothermal activity toward the surface and the insoluble fraction being stored in HP ultramafic clasts.

Despite the fact that abundant carbonate minerals may be sampled in the shallow Mariana forearc, as late calcite and aragonite veins, chimneys (*49*–*51*), or stored within blue-serpentinite (this study), their carbon isotope composition suggest that they formed nearby the surface through microbially mediated fluid interaction between shallow seawater and deep-sourced fluids. It is therefore not clear how much of this carbon is derived from the subduction process and how much is derived from late seawater circulation. Hence, these authigenic carbonates provide few constraints on deep carbon transfers between the slab and the mantle wedge. In contrast, the Atg-serpentinites preserve a deep geochemical signature reflecting CO_2_ transfer and reduction during the serpentinization of the forearc (Fig. 4). In these samples, the solid organic carbon fraction dominates over the inorganic carbon fraction, the former representing up to 80% of stored carbon.

Using our data in combination with recent estimates of total water flux in the Mariana subduction zone (*65*), we can provide a semiquantitative estimate of the maximum amount of solid organic carbon stored during forearc serpentinization (table S4). This calculation considers that between 20 and 74% of total water injected at the trench is transferred to the forearc mantle wedge by slab-derived fluids (*66*, *67*), where it is stored in serpentine minerals during peridotite serpentinization. The amount of carbon stored as solid organic compounds is then considered constant and constrained by the molar abundance of C_TOC_ relative to H_2_O in Atg-serpentinites (table S4). Using this calculation, we can propose that between 0.05 and 0.39 megatons year^−1^ of solid organic carbon is currently formed and stored in the Mariana forearc area. This represents between 5 and 54% of the carbon input of the Mariana subduction zone according to recent estimates of subducting sediments input by Clift (*68*) and Sadofsky and Bebout (*57*). Our estimate of the organic carbon input serves to underline the potential of the serpentinized forearc mantle as a major reservoir of solid organic carbon in subduction zones. The fate of this solid organic carbon reservoir needs further investigation. It may be transferred to the surface during clast and mud exhumation at the seafloor where it could serve as a carbon source for microbial ecosystems (*32*, *36*). If so, the low δ^13^C_TIC_ (<< 10‰; Fig. 4A) of the blue-serpentinites may reflect the microbial conversion of deep carbonaceous matter to authigenic carbonate. On the other hand, solid organic carbon may accumulate in the forearc along subduction until its transfer is operated to greater depths by mantle convection (*69*) or to the surface by plate tectonic (e.g., orogeny). The stability and devolatilization of such a solid organic carbon reservoir will then be intimately related to sharp changes of *f*O_2_ and pH over a large range of pressure and temperature (up to 6 GPa and 1000°C) (*21*, *31*).

## MATERIALS AND METHODS

The IODP identification numbers, bulk and in situ major (including bulk Fe^3+^/ΣFe quantification), and trace element chemistry of the studied samples are detailed in (*36*, *37*).

### Magnetic properties

Density and magnetic susceptibility measurements were performed on 21 clasts from Yinazao, 37 clasts from Fantangisña, and 31 clasts from Asùt Tesoru mud volcanoes (table S2). Analyzed sample volume was between 5 and 15 cm^3^. Measurements were performed either offshore during the IODP Expedition 366 (*35*) or onshore at the University of Lorraine (Nancy, France), using the same analytical devices and protocols. Density measurements were carried out by constant-volume gas pycnometry, using a Micromeritics AccuPyc II 1340 helium pycnometer at room temperature with a testing chamber of 3.5 cm^3^. Bulk magnetic susceptibility was measured at room temperature with an MFK1-A Kappabridge instrument (constant magnetizing field and operating frequency) based on the precise sample volume measurements from gas pycnometry analyses. A subset of 15 clasts with different mineralogy (i.e., blue-, Liz-, and Atg-serpentinites) was then selected for heat- and cool-induced magnetic transition measurements using Kappabridge devices CS-L Cryostat and CS-4 Furnace at the CRPG (Nancy, France). Measurements were performed under argon atmosphere at three temperature intervals: from −194° to 20°C using liquid nitrogen, from 20° to 700°C (heating curve), and from 700° to 20°C (cooling curve).

### X-ray CT

X-ray CT scanning of four samples with different mineralogy (i.e., blue-, Liz-, and Atg-serpentinites) was performed using a Phoenix Nanotom X-ray CT scanner at the University of Lorraine, GeoRessources. Scans were performed at 90 keV and 125 mA, with a voxel size of 5 to 6 μm^3^. Scans were reconstructed with Avizo software using a filtered rear projection method and a beam hardening correction. Reconstructed volumes were saved as tiff 16-bit uncompressed images.

### Sample preparation for carbon imaging

Sample preparation for organic carbon characterization was performed at the Institut de physique du globe de Paris (IPGP, France). Drilled rock samples were sawed with sterile ultrapure water to extract the inner core, free of possible postsampling contamination. The saw was previously rinsed twice with sterile ultrapure water. The inner core was then manipulated using clean pliers, thinned, and polished on both faces (down to a thickness of 10 μm) with pure ethanol using silicon carbide polishing disks without any use of resin or glue.

### FTIR microimaging

FTIR microspectroscopy was performed at IPGP on a Thermo Fisher Scientific iN10 MX microscope (Ever-Glo conventional infrared source) equipped with a 15× objective (numerical aperture = 0.7) and a liquid nitrogen–cooled MCT-A detector. The incident beam was collimated to a sample area of 20 μm by 20 μm. The thinned rock sample was deposited on a BaF_2_ window without any treatment. FTIR hyperspectral maps were acquired in transmission in the range 4000 to 675 cm^−1^ using step size of 20 μm, and 64 accumulations per spectrum per pixel and the spectral resolution was set to 8 cm^−1^. Spectrum analyses and reconstructions based on band areas were performed using OMNIC software (Thermo Fisher Scientific). A second derivative was applied to raw data to remove baseline variations using the Stavitsky-Golay method (polynomial order, 2; filter width, 15). Second derivative maps were obtained from unit vector–normalized maps using MATLAB and PLS_Toolbox software (Eigenvector Research Inc.). Curve fitting in the absorbance region of the aliphatic moieties (i.e., 2800 to 3000 cm^−1^) was computed with Wire 3.2 software (Renishaw). Five mixed Lorentzian/Gaussian functions were used to account for all the symmetric and asymmetric C─H stretching bands. The methyl-to-methylene ratio (*R*_CH2/CH3_) was computed as the area ratio of the fitted asymmetric C─H stretching bands of CH_2_ to CH_3_ at ~2930/2960 cm^−1^, respectively. *R*_CH2/CH3_ allows rough assessment of the length and degree of branching of side groups associated with aliphatic chains (*70*).

### Scanning electron microscopy

SEM observations were performed at IPGP using a Zeiss Auriga FEG-FIB field emission scanning electron microscope. Samples were Au-coated. Images were collected using backscattered electron and secondary electron detector at high and low current, respectively, with accelerating voltage ranging from 10 to 15 kV. EDS measurements were performed at 15-kV accelerating voltage using a Bruker detector.

### Carbon bulk rock analyses

The TC concentrations (C_TC_) and isotope ratios (δ^13^C_TC_) analyses were performed using a Thermo Fisher Scientific EA IsoLink IRMS System at the CRPG (Nancy, France). Two to three aliquots of 30 mg of each powdered samples were wrapped in tin capsules and then burned at 1020°C by flash combustion in a quartz reactor filled with chromium oxide, pure copper, and silvered cobalt oxide. Produced gases (N_2_ and CO_2_) were separated on a chromatographic column maintained at 70°C, and TC concentrations and isotope ratios were then measured using a Thermo Fisher Scientific Delta V Advantage continuous flow isotope ratio mass spectrometer (IRMS). TC isotopic composition was determined by comparison with CRPG and international standards that were analyzed along with samples: (i) δ^13^C_TC (BFSd)_ = −21.48‰; (ii) δ^13^C_TC(CRPG_M2)_ = −24.97‰; and (iii) δ^13^C_TC (PSd)_ = −22.58‰. Values are quoted in the delta notation in ‰ relative to the V-PDB (Vienna PeeDee Belemnite) standard, and the reproducibility is better than 0.2‰. Internal standards were used to determine carbon concentrations: (i) [C_TC (BFSd)_] = 0.52 wt %; (ii) [C_TC (CRPG_M2)_] = 0.41 wt %. For each sample, the 2σ error is expected to be lower than 5% for (C_TC_) and 1‰ for δ^13^C_TC_. TOC concentrations (C_TOC_) and isotopic compositions (δ^13^C_TOC_) were determined for each whole-rock sample using the same instruments and calibration described for analyses of the TC. The inorganic carbon component was removed, before each analysis, by HCl fumigation during 5 days at 65°C.

TIC isotope ratios (δ^13^C_TIC_) were determined by using an auto sampler Gasbench coupled to a Thermo Fisher Scientific MAT 253 continuous flow IRMS at the CRPG (Nancy, France). For each sample, an aliquot of 1 to 100 mg of powder was reacted with 2 ml of supersaturated orthophosphoric acid at 70°C for 10 hours under a He atmosphere. Values are quoted in the delta notation in ‰ relative to V-PDB. All sample measurements were adjusted to an internal reference (MCt: pure calcite; δ^13^C_TIC_ = −8.64‰) calibrated on international standards (i) δ^13^C_TIC (BR516)_ = −1.1‰ and (ii) δ^13^C_TIC (BR8107)_ = −0.9‰. The reproducibility of the standards was better than 0.2‰. TIC contents (C_TIC_) of the samples were determined by comparison with two internal standards consisting in fine-grained marine sediments from the Bay of Bengal and routinely included during the analysis: (i) [CaCO_3 (BR516)_] = 3.54 wt % and (ii) [CaCO_3 (BR8107)_] = 6.16 wt %. The 2σ errors for carbonate contents are estimated to be lower than 5%.

## Supplementary Materials

### This PDF file includes:

Figs. S1 to S6 Tables S1 to S4 References
