# Supplementary material for: High-pressure synthesis and storage of solid organic compounds in active subduction zones
Source: Sci Adv. 2022 Sep 16;8(37):eabo2397. doi: 10.1126/sciadv.abo2397 (PMC9481122; doi:10.1126/sciadv.abo2397)
Supplement: Supplementary file 1 — Figs. S1 to S6 Tables S1 to S4 References [file sciadv.abo2397_sm.pdf]

Science Advances
AAAS

Supplementary Materials for

High-pressure synthesis and storage of solid organic compounds in active subduction zones

Baptiste Debret *et al.*

Corresponding author: Baptiste Debret, debret@ipgp.fr

Sci. Adv. **8**, eabo2397 (2022)
DOI: 10.1126/sciadv.abo2397

This PDF file includes:

Figs. S1 to S6
Tables S1 to S4
References

Supplementary Materials

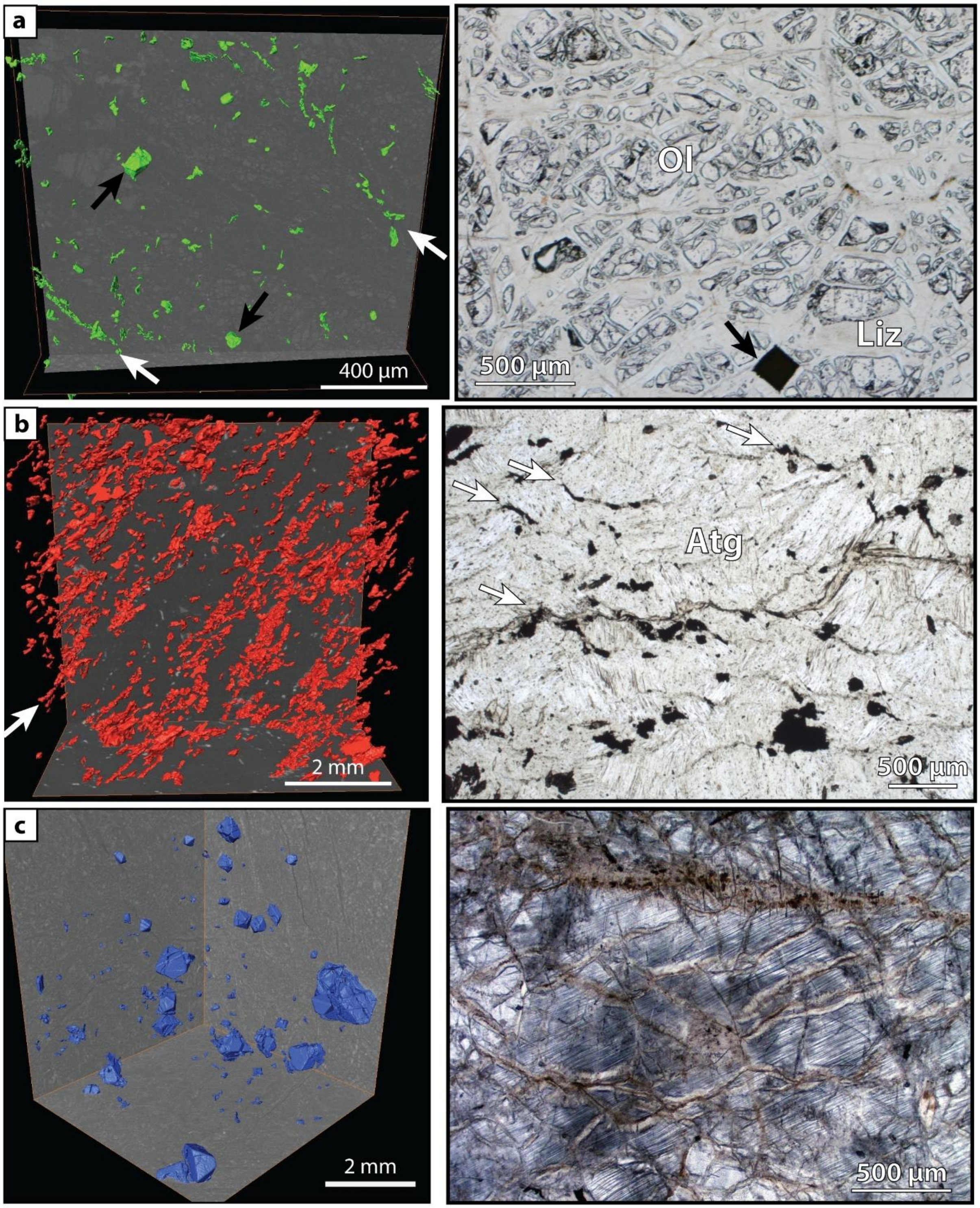

Fig. S1. **Reconstructed 3D CT images and associated plane polarized light thin section microphotographs of (a) a Liz-, (b) an Atg- and (c) a blue-serpentinite.** It reveals contrasted Fe-bearing opaque mineral distribution as temperature and pressure evolve in the Marianna forearc. **a** The Liz-serpentinite is mainly composed of brown serpentine (i.e., lizardite and/or chrysotile) and Fe-rich brucite bearing veins forming mesh textures after olivine (Ol). It preserved two generations of spinels: pyramidal mantle spinels randomly distributed within the rock (black arrows) and thin orientated magnetite veins (white arrows, not shown on the thin section microphotograph). **b** The Atg-serpentinite is fully recrystallized into antigorite needles associated with large veins of magnetite and Fe-poor brucite (white arrows) orientated along preferential plans. **c** The blue-serpentinites consist mainly of blue serpentine, mantle Al-Cr-Mg-Fe

spinel and sulfides with rare hydrogrossular and relicts of mantle olivine. Mantle spinels display euhedral shapes and are easily distinguishable from large pyramidal sulfides on the CT scan. Details on serpentinization timing and processes in relation to fluid circulation and mantle flow within the Mariana forearc can be found in the main text.

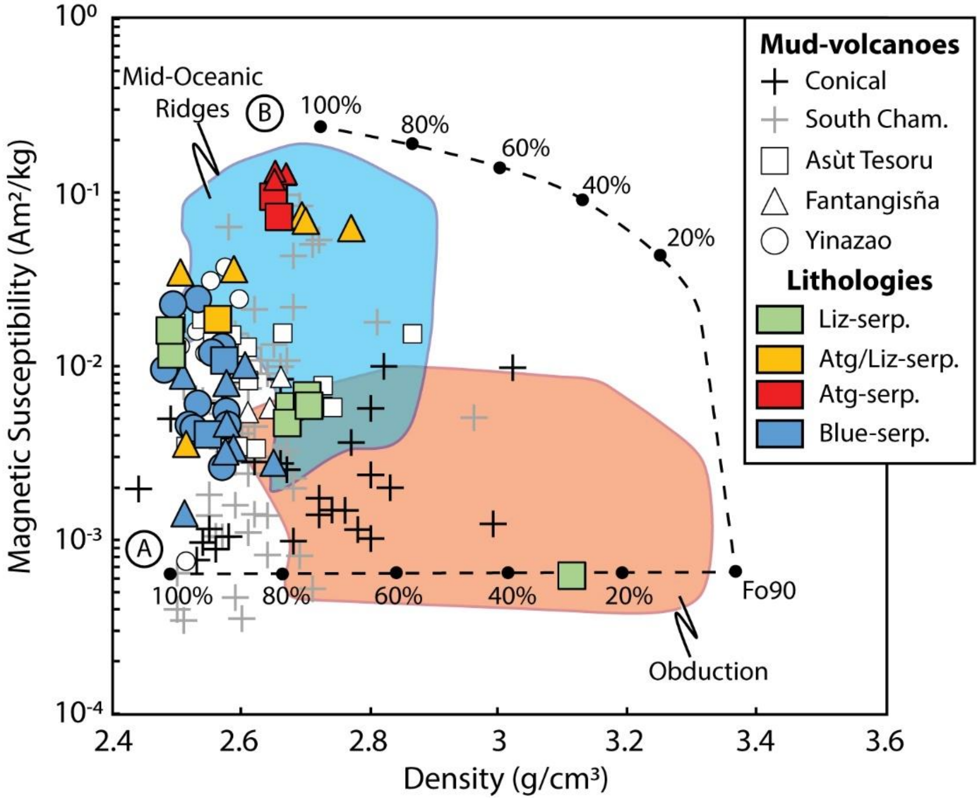

Fig. S2. **Bulk magnetic susceptibility versus density of serpentinized ultramafic clasts (serp.) from the Mariana forearc.** The two endmember trend paths are from ref. (*73*); they represent the low temperature serpentinization reaction with no magnetite formation (curve A) and the high temperature serpentinization reaction with abundant magnetite precipitation (curve B). Serpentinization degree increments of 20% (starting from Fo_{90} olivine) are indicated along the curves by black dots and labelled. The coloured shaded areas correspond to the fields of abyssal (in blue) and obduction related (in red) serpentinites (modified after *49*). Bulk magnetic susceptibility measurements of clasts recovered during previous IODP and ODP expeditions at South Chamorro and Conical mud volcanoes (*74*, *75*) are shown for comparison.

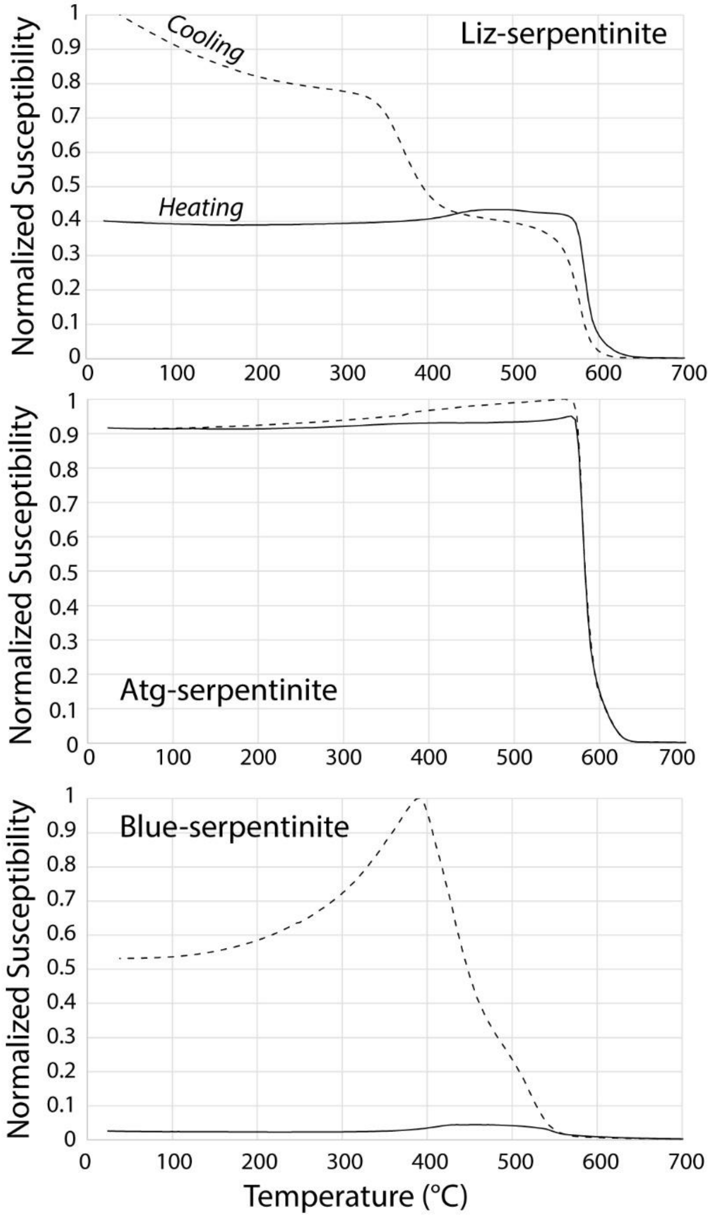

Fig. S3. Representative mass normalized thermomagnetic curves for Liz-, Atg- and blue- serpentinites. The Liz-and blue-serpentinites show an irreversible behaviour during successive heating (black line) and cooling (dashed line) of the sample, with the final susceptibility being higher at the end of the cooling cycle. In the case of Liz-serpentinites, the irreversible behaviour of thermomagnetic curves is attributed to the formation of pyrrhotite (Curie temperature between 350-440°C) during the heating, possibly related to greigite decomposition (*76*). In the case of blue-serpentinite, similar processes can explain the high increase of thermomagnetic curves during the cooling. The sudden drop of magnetic susceptibility at low temperature (< 400°C) reflects the presence of a large amount of sulfides and/or sulfate favouring both magnetite, pyrrhotite and greigite formation along sample heating (*76*). The Atg-serpentinites display a reversible behaviour during sample heating and cooling, indicating that no mineralogical changes occurred during the heating. The inflexion point is always between 575°C and 585°C showing that magnetite is the dominant magnetic carrier in these samples.

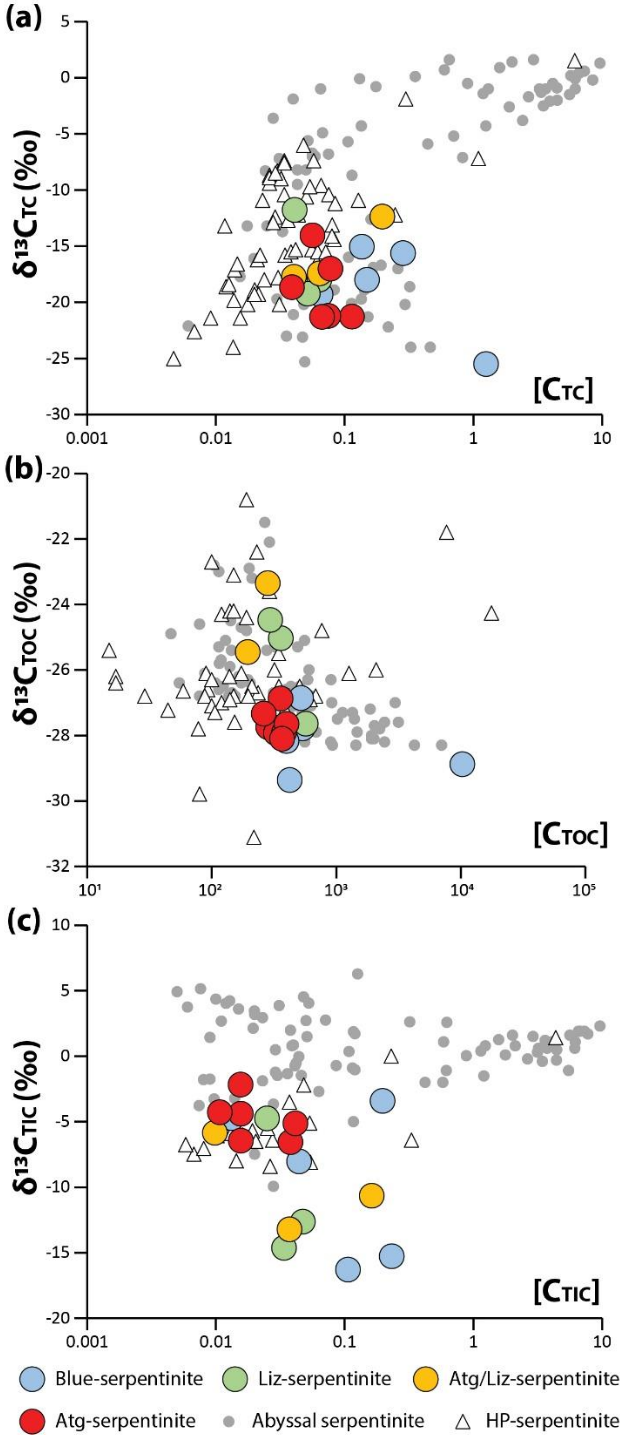

Fig. S4. Carbon concentrations (TC: Total Carbon, TOC: Total Organic Carbon; TIC: Total Inorganic Carbon from (a) to (c), respectively) and isotope variations in Mariana serpentinized clasts compared with worldwide serpentinite data. Abyssal serpentinite and HP-serpentinite data are from (*42*, *43*) and (*44*, *45*), respectively. Note that the $\delta^{13}C_{TIC}$ of abyssal serpentinites with low $[C_{TIC}]$ (< 400 ppm) was calculated considering $\delta^{13}C_{TC}$, $\delta^{13}C_{TOC}$, $[C_{TC}]$ and $[C_{TOC}]$ analyses as no data are available.

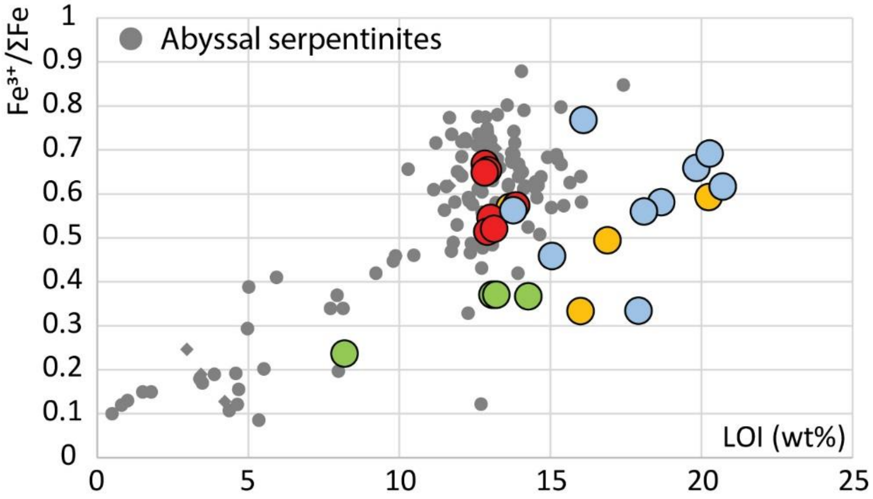

Fig. S5. Bulk serpentinites Loss On Ignition (LOI; in wt%) versus $Fe^{3+}/\Sigma Fe$ ratios. Figure modified after (*37*).

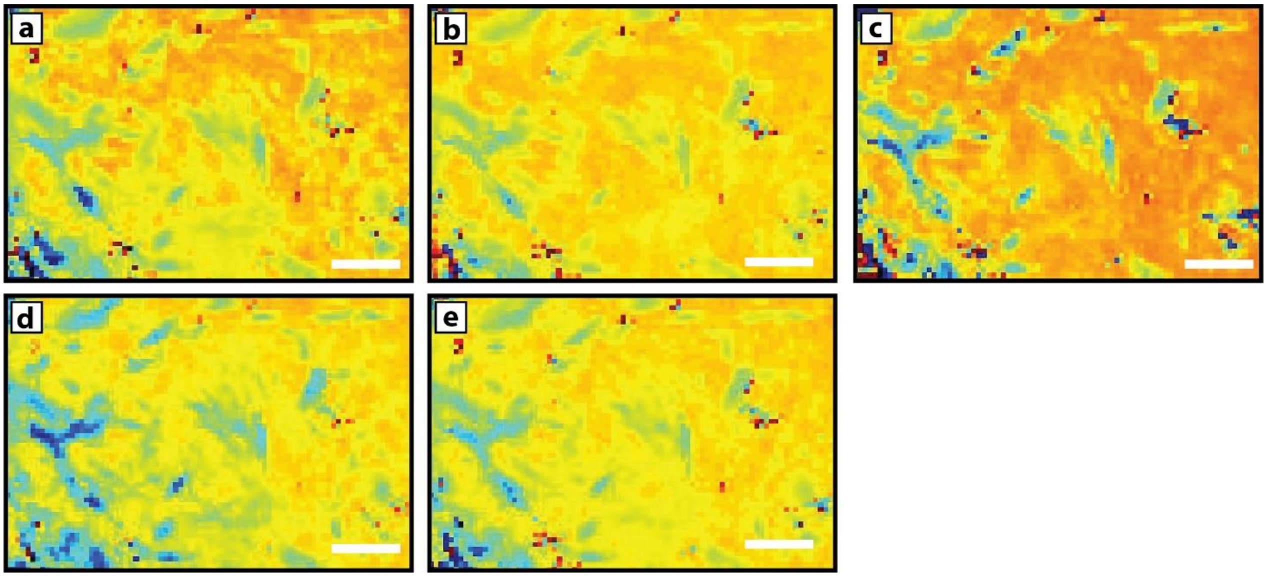

Fig. S6. FTIR map obtained from signal integration at various wavenumber. Maps were integrated at **a** 2965 cm^{-1} (CH_3 asymmetric C-H stretching), **b** 2923 cm^{-1} (CH_2 asymmetric C-H stretching), **c** 1732 cm^{-1} (C=O stretching of aliphatic aldehyde), **d** 1462 cm^{-1} (CH_3 asymmetric C-H bending, CH_2 scissoring), **e** 1383 cm^{-1} (CH_3 symmetric C-H bending) considering second derivative spectra for the hyperspectral map shown in Fig. 2. Colour scale is opposite compared to the latter (i.e., blue and red colours depict highest and lowest absorbance, respectively). Scale bar is 300 μm.

Sample Name	Seamount	Label identifier	$Fe^{3+}/\Sigma Fe$	LOI (wt%)	As (ppm)	B (ppm)	$\delta^{56}Fe$ (‰)	2sd
Blue Serpentinites:								
M5	*Yinazao*	366-U1492C-5F-2-W 25/28	0.33	17.9	n.d.	7	0.05	0.03
M24	*Asùt Tesoru*	366-U1496B-5F-1-W 77/82	0.56	13.8	0.84	10.7	-0.02	0.04
M30	*Fantangisña*	366-U1497A-3G-CC-W 9/10	0.62	20.7	0.71	93	-0.01	0.05
M38	*Fantangisña*	366-U1498B-3R-3-W 89/92	0.58	18.7	0.57	18.7	n.d.	n.d.
M45	*Fantangisña*	366-U1498B-13R-1-W 37/45	0.46	15.1	1.85	26.7	-0.04	0.02
M6	*Yinazao*	366-U1492C-5F-3-W 43/47	0.66	19.9	0.50	6.7	-0.07	0.05
M2	*Yinazao*	366-U1492A-1H-2-W 139/140	0.77	16.1	0.63	240	0.00	0.02
M3	*Yinazao*	366-U1492A-1H-3-MBIO2(115-135)	0.69	20.3	1.02	165	0.05	0.01
M7	*Yinazao*	366-U1492C-12F-2-W 19/22-TSB-TS_37	0.56	18.1	1.05	7.9	-0.01	0.02
Liz-Serpentinites:								
TS55	*Asùt Tesoru*	366-U1493B-9X-1-W 56/58-TSB-TS_55	0.24	8.2	1.26	20.7	-0.12	0.03
M9	*Asùt Tesoru*	366-U1493B-9X-CC-W 4/9	0.37	13.1	0.66	29.7	0.09	0.02
M10	*Asùt Tesoru*	366-U1493B-9X-CC-W 14/16	0.37	13.2	0.84	29.5	-0.03	0.03
M19	*Asùt Tesoru*	366-U1496A-1F-1-W 6/8	0.37	14.3	n.d.	29.6	n.d.	n.d.
Atg/Liz-Serpentinites:								
M12	*Asùt Tesoru*	366-U1495B-3G-CC-W 5/7	0.59	20.3	1.81	58.5	-0.21	0.05
M13	*Asùt Tesoru*	366-U1495B-3G-CC-W 24/26	0.33	16.0	1.33	47.7	0.00	0.02
M32	*Fantangisña*	366-U1497A-13G-CC-W 52/55	0.57	13.7	1.94	48	-0.04	0.02
M27	*Asùt Tesoru*	366-U1496B-10F-1-W 34/40	0.49	16.9	0.84	17.6	n.d.	n.d.
Atg-Serpentinites:								
M14	*Asùt Tesoru*	366-U1495B-5G-CC-W 17/20	0.57	13.9	1.47	16	-0.05	0.05
M15	*Asùt Tesoru*	366-U1495B-6F-CC-W 10/12	0.54	13.0	3.28	9.8	0.00	0.03
M16	*Asùt Tesoru*	366-U1495A-3G-CC-W 13/15-TSB-TS_63	0.51	12.9	4.39	39.4	-0.09	0.01
M17	*Asùt Tesoru*	366-U1495A-4F-1-W 86/89-TSB-TS_65	0.52	13.1	2.71	48.6	-0.08	0.02
M50	*Fantangisña*	366-U1497B-4G-1-W 3/5	0.67	12.8	7.85	95	-0.14	0.00
M51	*Fantangisña*	366-U1497B-4G-1-W 8/12	0.65	12.9	7.62	94.7	-0.23	0.01
TSB102	*Fantangisña*	366-U1497B-4G-1-W 3/6-TSB-TS_102	0.65	12.8	9.17	96.7	-0.26	0.06

Table S1. Fe redox state and isotopes, Loss On Ignition (LOI) and fluid mobile element (As and B) analyses of Mariana forearc serpentinites. n.d. not determined; sd: standard deviation. Analyses are from (*36*, *37*)

Site	*Hole*	*Core*	*Type*	*Section*	*Offset (cm)*	*Depth (mbsf)*	*Density (g/cm³)*	*Bulk Ms (SI)*	*Ms (Am2/kg)*	*%Magnetite*	**Serp. degree**
U1492	A	1	H	1	139	1.39	2.573	1.27E-02	0.53	0.58	0.95
U1492	A	1	H	2	137	2.88	2.535	5.97E-03	0.25	0.27	0.98
U1492	A	1	H	3	115	4.17	2.483	9.38E-03	0.39	0.43	1.06
U1492	B	2	F	1	145	8.55	2.517	7.58E-04	0.03	0.03	1.00
U1492	B	3	F	2	115	14.06	2.579	5.39E-03	0.23	0.25	0.93
U1492	B	3	F	2	115	14.06	2.522	4.52E-03	0.19	0.21	1.00
U1492	B	3	F	2	120	14.11	2.529	4.34E-03	0.18	0.20	0.99
U1492	B	4	F	2	20	17.39	2.508	1.30E-02	0.54	0.59	1.03
U1492	B	4	F	2	89	18.08	2.581	4.51E-03	0.19	0.21	0.92
U1492	B	6	F	1	129	26.39	2.535	2.38E-02	1.00	1.08	1.01
U1492	C	1	H	4	18	4.66	2.557	1.17E-02	0.49	0.53	0.96
U1492	C	5	F	1	52	14.72	2.599	2.41E-02	1.01	1.10	0.93
U1492	C	5	F	1	126	15.46	2.579	1.22E-02	0.51	0.55	0.94
U1492	C	5	F	2	25	15.95	2.573	2.60E-03	0.11	0.12	0.93
U1492	C	5	F	3	43	17.63	2.497	2.21E-02	0.93	1.01	1.06
U1492	C	14	F	2	35	53.55	2.555	3.07E-02	1.28	1.40	1.00
U1492	C	19	F	3	0	77.55	2.577	3.68E-02	1.54	1.67	0.98
U1492	C	26	F	1	62	108.72	2.533	1.58E-02	0.66	0.72	1.00
U1492	C	26	F	1	72	108.82	2.546	1.18E-02	0.49	0.54	0.98
U1492	C	28	G	1	18	117.68	2.576	1.02E-02	0.43	0.47	0.94
U1493	A	4	F	2	73	7.83	2.501	1.75E-02	0.73	0.80	1.05
U1493	B	3	G	CC	21	5.31	2.541	1.85E-02	0.77	0.84	1.00
U1493	B	9	X	1	56	30.16	2.866	1.53E-02	0.64	0.69	0.57
U1493	B	9	X	1	74	30.34	2.611	1.28E-02	0.53	0.58	0.90
U1493	B	9	X	CC	4	30.57	2.676	5.77E-03	0.24	0.26	0.80
U1493	B	9	X	CC	6	30.59	2.728	7.67E-03	0.32	0.35	0.74
U1493	B	9	X	CC	14	30.67	2.703	6.66E-03	0.28	0.30	0.77
U1493	B	9	X	CC	19	30.72	2.587	1.50E-02	0.63	0.68	0.93
U1493	B	9	X	1	68	-	2.743	5.76E-03	0.24	0.26	0.72
U1495	A	4	F	1	86	6.86	2.652	9.20E-02	3.85	4.18	0.97
U1495	B	3	G	CC	5	4.84	2.495	1.15E-02	0.48	0.52	1.04
U1495	B	3	G	CC	24	5.03	2.492	1.57E-02	0.66	0.72	1.05
U1496	A	1	F	1	6	0.06	2.673	4.63E-03	0.19	0.21	0.81
U1496	A	1	F	1	120	1.20	2.579	3.37E-03	0.14	0.15	0.92
U1496	A	3	F	5	28	11.71	2.558	1.36E-02	0.57	0.62	0.97
U1496	A	3	F	5	36	11.79	2.576	1.06E-02	0.44	0.48	0.94
U1496	A	5	F	CC	29	21.83	2.666	1.53E-02	0.64	0.70	0.83
U1496	A	6	F	2	94	23.27	2.551	3.97E-03	0.17	0.18	0.96
U1496	A	6	F	3	79	24.11	2.613	8.22E-03	0.34	0.37	0.89
U1496	A	7	F	2	47	28.60	2.597	3.44E-03	0.14	0.16	0.90
U1496	A	10	G	CC	18	40.68	3.111	6.16E-04	0.03	0.03	0.24
U1496	B	2	F	1	72	2.62	2.517	3.44E-03	0.14	0.16	1.00
U1496	B	5	F	1	81	16.81	2.660	7.04E-02	2.95	3.20	0.92
U1496	B	5	F	1	122	17.22	2.625	3.34E-03	0.14	0.15	0.86
U1496	B	10	F	1	34	34.74	2.566	1.82E-02	0.76	0.83	0.96
U1496	C	5	R	1	8	36.68	2.706	5.83E-03	0.24	0.26	0.76
U1496	C	5	R	1	15	36.75	2.711	6.08E-03	0.25	0.28	0.76
U1497	A	2	F	1	80	1.70	2.517	3.52E-03	0.15	0.16	1.00

U1497	A	2	F	2	60	3.01	2.580	1.39E-02	0.58	0.63	0.94
U1497	A	3	G	CC	9	5.53	2.391	2.00E-03	0.08	0.09	1.16
U1497	A	4	X	CC	13	5.73	2.580	4.59E-03	0.19	0.21	0.92
U1497	A	4	X	CC	14	5.74	2.596	9.25E-03	0.39	0.42	0.91
U1497	A	5	F	4	16	16.26	2.646	5.75E-03	0.24	0.26	0.84
U1497	A	5	F	4	47	16.57	2.663	8.65E-03	0.36	0.39	0.82
U1497	A	8	F	2	14	25.29	2.590	3.35E-03	0.14	0.15	0.91
U1497	A	9	G	CC	21	28.26	2.612	5.42E-03	0.23	0.25	0.88
U1497	A	13	G	CC	50	34.35	2.695	7.20E-02	3.01	3.28	0.88
U1497	A	13	G	CC	52	34.37	2.701	6.71E-02	2.81	3.05	0.86
U1497	A	13	G	CC	61	34.46	2.771	6.08E-02	2.54	2.77	0.76
U1497	B	4	G	1	3	12.88	2.671	1.25E-01	5.21	5.66	0.99
U1497	B	4	G	1	3	12.88	2.654	1.29E-01	5.39	5.86	1.02
U1497	B	4	G	1	9	12.94	2.653	1.16E-01	4.83	5.25	1.00
U1498	A	3	R	2	37	16.49	2.505	6.08E-03	0.25	0.28	1.02
U1498	A	3	R	2	52	16.64	2.554	3.73E-03	0.16	0.17	0.96
U1498	A	3	R	3	13	17.12	2.591	3.54E-02	1.48	1.61	0.96
U1498	A	3	R	3	6	17.52	2.508	3.40E-02	1.42	1.55	1.06
U1498	B	3	R	1	76	19.96	2.519	4.39E-03	0.18	0.20	1.00
U1498	B	3	R	3	89	21.29	2.608	9.97E-03	0.42	0.45	0.90
U1498	B	3	R	3	15	20.55	2.512	8.66E-03	0.36	0.39	1.02
U1498	B	3	R	3	71	21.11	2.509	1.86E-03	0.08	0.08	1.01
U1498	B	4	R	2	16	30.18	2.560	5.15E-03	0.22	0.23	0.95
U1498	B	4	R	2	36	30.38	2.532	4.82E-03	0.20	0.22	0.99
U1498	B	4	R	2	66	30.68	2.509	4.05E-03	0.17	0.18	1.01
U1498	B	5	R	1	59	35.29	2.560	3.70E-03	0.15	0.17	0.95
U1498	B	5	R	1	69	35.39	2.539	9.12E-03	0.38	0.41	0.98
U1498	B	5	R	2	34	36.43	2.525	3.51E-03	0.15	0.16	0.99
U1498	B	6	R	1	39	44.79	2.515	1.41E-03	0.06	0.06	1.00
U1498	B	6	R	1	111	45.51	2.604	6.05E-03	0.25	0.28	0.90
U1498	B	6	R	1	121	45.61	2.567	2.75E-03	0.12	0.13	0.94
U1498	B	7	R	4	63	57.22	2.552	4.21E-03	0.18	0.19	0.96
U1498	B	7	R	4	84	57.43	2.567	6.02E-03	0.25	0.27	0.94
U1498	B	7	R	4	115	57.74	2.551	3.86E-03	0.16	0.18	0.96
U1498	B	8	R	3	3	65.26	2.591	6.20E-03	0.26	0.28	0.91
U1498	B	9	R	1	37	73.97	2.578	3.18E-03	0.13	0.14	0.92
U1498	B	9	R	1	39	73.99	2.608	3.71E-03	0.16	0.17	0.89
U1498	B	10	R	2	21	85.00	2.576	4.96E-03	0.21	0.23	0.93
U1498	B	12	R	2	140	105.70	2.564	2.74E-03	0.11	0.12	0.94
U1498	B	13	R	1	37	112.87	2.579	7.87E-03	0.33	0.36	0.93
U1498	B	13	R	3	93	114.56	2.652	2.74E-03	0.11	0.12	0.83
U1498	B	13	R	3	109	114.72	2.652	2.79E-03	0.12	0.13	0.83
U1498	B	18	R	2	17	-	2.544	1.62E-03	0.07	0.07	0.97
U1498	B	18	R	2	19	161.64	2.523	5.19E-03	0.22	0.24	1.00
U1498	B	23	R	3	25	-	2.543	4.57E-03	0.19	0.21	0.97

Table S2. Density, magnetic susceptibility (Ms) and magnetite modal percentage (%Magnetite) of the recovered ultramafic clasts from Yinazao, Asùt Tesoru and Fantangisña mud-volcanoes. Magnetite modal percentage is calculated from the magnetic susceptibility values (see (*73*) for more details). mbsf stands for meter below seafloor.

Sample Name	Seamount	Label identifier	$[C_{TC}]$(wt%)	$\delta^{13}C_{TC}$ (‰)	$[C_{TIC}]$	$\delta^{13}C_{TIC}$	$[C_{TOC}]$	$\delta^{13}C_{TOC}$	% TIC
Blue Serpentinites:									
M5	*Yinazao*	366-U1492C-5F-2-W 25/28	0.065	-19.3	0.013	-4.7	0.052	-26.9	20
M24	*Asùt Tesoru*	366-U1496B-5F-1-W 77/82	n.d.	n.d.	n.d.	n.d.	n.d.	n.d.	
M30	*Fantangisña*	366-U1497A-3G-CC-W 9/10	1.252	-25.5	0.197	-3.4	1.029	-26.9	16
M38	*Fantangisña*	366-U1498B-3R-3-W 89/92	0.284	-15.6	0.232	-12.8	0.054	-27.8	81
M45	*Fantangisña*	366-U1498B-13R-1-W 37/45	0.149	-18.0	0.106	-14.2	0.040	-28.2	73
M6	*Yinazao*	366-U1492C-5F-3-W 43/47	n.d.	n.d.	n.d.	n.d.	n.d.	n.d.	
M2	*Yinazao*	366-U1492A-1H-2-W 139/140	n.d.	n.d.	n.d.	n.d.	n.d.	n.d.	
M3	*Yinazao*	366-U1492A-1H-3-MBIO2(115-135)	n.d.	n.d.	n.d.	n.d.	n.d.	n.d.	
M7	*Yinazao*	366-U1492C-12F-2-W 19/22-TSB-TS_37	0.136	-15.0	0.044	-8.0	0.042	-29.4	51
Liz-Serpentinites:									
TS55	*Asùt Tesoru*	366-U1493B-9X-1-W 56/58-TSB-TS_55	0.041	-11.8	0.025	-4.7	0.057	-27.6	31
M9	*Asùt Tesoru*	366-U1493B-9X-CC-W 4/9	0.064	-18.0	0.047	-12.6	0.036	-25.0	57
M10	*Asùt Tesoru*	366-U1493B-9X-CC-W 14/16	0.052	-19.2	0.034	-14.6	0.030	-24.5	53
M19	*Asùt Tesoru*	366-U1496A-1F-1-W 6/8	n.d.	n.d.	n.d.	n.d.	n.d.	n.d.	
Atg/Liz-Serpentinites:									
M12	*Asùt Tesoru*	366-U1495B-3G-CC-W 5/7	0.196	-12.4	0.162	-10.6	0.028	-23.3	85
M13	*Asùt Tesoru*	366-U1495B-3G-CC-W 24/26	0.064	-17.4	0.037	-13.2	0.019	-25.5	66
M32	*Fantangisña*	366-U1497A-13G-CC-W 52/55	0.040	-17.7	0.010	-5.9	0.031	-27.7	24
M27	*Asùt Tesoru*	366-U1496B-10F-1-W 34/40	n.d.	n.d.	n.d.	n.d.	n.d.	n.d.	
Atg-Serpentinites:									
M14	*Asùt Tesoru*	366-U1495B-5G-CC-W 17/20	0.056	-14.0	0.038	-6.6	0.028	-27.7	57
M15	*Asùt Tesoru*	366-U1495B-6F-CC-W 10/12	0.039	-18.7	0.016	-6.4	0.033	-27.9	32
M16	*Asùt Tesoru*	366-U1495A-3G-CC-W 13/15-TSB-TS_63	0.114	-21.3	0.016	-4.4	0.038	-27.9	29
M17	*Asùt Tesoru*	366-U1495A-4F-1-W 86/89-TSB-TS_65	0.078	-17.0	0.041	-5.1	0.040	-27.7	51
M50	*Fantangisña*	366-U1497B-4G-1-W 3/5	0.074	-21.2	0.011	-4.3	0.037	-28.1	23
M51	*Fantangisña*	366-U1497B-4G-1-W 8/12	n.d.	n.d.	n.d.	n.d.	n.d.	n.d.	
TSB102	*Fantangisña*	366-U1497B-4G-1-W 3/6-TSB-TS_102	0.067	-21.3	0.016	-2.2	0.026	-27.3	37

Table S3. Total (TC), inorganic (TIC) and organic (TOC) carbon concentration, associated relative proportion (%TIC) and isotope analyses of serpentinites. n.d.: not determined.

Flux	H_2O Input[a]	H_2O transferred to the mantle wedge[b]	C_{TOC} stored[c]	C_{TOC} stored[d]	C input[e]
	(Mt.yr^{-1}.km^{-1})	(Mt.yr^{-1}.km^{-1})	(Mt.yr^{-1}.km^{-1})	(Mt.yr^{-1})	(Mt.yr^{-1})
max	1.1E-01	8.2E-02	3.7E-04	4.0E-01	9.8E-01
min	7.7E-02	1.5E-02	4.8E-05	7.4E-02	7.2E-01

Table S4. Estimated annual sedimentary C_{TOC} storage at the Mariana forearc.

a Water maximum and minimum fluxes to Mariana trench estimated by Cai et al. (*65*) based on seismic images of the crust and uppermost mantle around the central Mariana trench.

b H_2O transferred to mantle wedge; the minimum value corresponds to 20% of the minimum H_2O input, i.e., the fraction of forearc mantle water that contributes to the water released beneath the arc (*67*), and the maximum value to 74 % of the maximum H_2O input, based on present-day conditions of the global influx water recycled into the mantle (*66*).

c The stored C_{TOC} is based on the molar ratio [C_{TOC}] / H_2O in Atg-serpentinites, which varies from 0.0031 (sample TSB102) to 0.0045 (sample M17; Tables S1 and S3).

d Carbon stored in the forearc over 1050 km linear trench length.

e Total carbon subducted at the Mariana trench; the maximum value is from Sadofky & Bebout (*57*) and the minimum value is from Clift (*68*).

REFERENCES AND NOTES

1. J. F. Kasting, D. H. Eggler, S. P. Raeburn, Mantle redox evolution and the oxidation state of the archean atmosphere. *J. Geol.* **101**, 245–257 (1993).

2. F. Gaillard, B. Scaillet, N. T. Arndt, Atmospheric oxygenation caused by a change in volcanic degassing pressure. *Nature* **478**, 229–232 (2011).

3. M. S. Duncan, R. Dasgupta, Rise of Earth's atmospheric oxygen controlled by efficient subduction of organic carbon. *Nat. Geosci.* **10**, 387–392 (2017).

4. V. Stagno, D. O. Ojwang, C. A. McCammon, D. J. Frost, The oxidation state of the mantle and the extraction of carbon from Earth's interior. *Nature* **493**, 84–88 (2013).

5. S. Poli, Carbon mobilized at shallow depths in subduction zones by carbonatitic liquids. *Nat. Geosci.* **8**, 633–636 (2015).

6. P. J. Gorman, D. M. Kerrick, J. A. D. Connolly, Modeling open system metamorphic decarbonation of subducting slabs. *Geochem. Geophys. Geosyst.* **7**, Q04007 (2006).

7. R. Dasgupta, M. M. Hirschmann, The deep carbon cycle and melting in Earth's interior. *Earth Planet. Sci. Lett.* **298**, 1–13 (2010).

8. P. B. Kelemen, C. E. Manning, Reevaluating carbon fluxes in subduction zones, what goes down, mostly comes up. *Proc. Natl. Acad. Sci. U.S.A.* **112**, E3997–E4006 (2015).

9. J. C. Alt, D. A. H. Teagle, The uptake of carbon during alteration of ocean crust. *Geochim. Cosmochim. Acta* **63**, 1527–1535 (1999).

10. T. Plank, The chemical composition of subducting sediments, in *Treatise on Geochemistry: Second Edition*, R. F. Keeling, Ed. (Elsevier, 2014), vol. 2, pp. 607–629.

11. J. C. Alt, E. M. Schwarzenbach, G. L. Früh-green, W. C. Shanks III, S. M. Bernasconi, C. J. Garrido, L. Crispini, L. Gaggero, J. A. Padrón-navarta, C. Marchesi, The role of serpentinites in cycling of

carbon and sulfur: Seafloor serpentinization and subduction metamorphism. *Lithos* **178**, 40–54 (2013).

12. S. Shilobreeva, I. Martinez, V. Busigny, P. Agrinier, C. Laverne, Insights into C and H storage in the altered oceanic crust: Results from ODP/IODP Hole 1256D. *Geochim. Cosmochim. Acta* **75**, 2237–2255 (2011).

13. D. V. Bekaert, S. J. Turner, M. W. Broadley, J. D. Barnes, S. A. Halldórsson, J. Labidi, J. Wade, K. J. Walowski, P. H. Barry, Subduction-driven volatile recycling: A global mass balance. *Annu. Rev. Earth Planet. Sci.* **49**, 37–70 (2021).

14. T. M. McCollom, J. S. Seewald, Abiotic synthesis of organic compounds in deep-sea hydrothermal environments. *Chem. Rev.* **107**, 382–401 (2007).

15. F. Klein, W. Bach, S. E. Humphris, W. A. Kahl, N. Jöns, B. Moskowitz, T. S. Berquó, Magnetite in seafloor serpentinite-Some like it hot. *Geology* **42**, 135–138 (2014).

16. M. Andreani, M. Muñoz, C. Marcaillou, A. Delacour, µXANES study of iron redox state in serpentine during oceanic serpentinization. *Lithos* **178**, 70–83 (2013).

17. N. G. Holm, J. L. Charlou, Initial indications of abiotic formation of hydrocarbons in the Rainbow ultramafic hydrothermal system, Mid-Atlantic Ridge. *Earth Planet. Sci. Lett.* **191**, 1–8 (2001).

18. B. Ménez, C. Pisapia, M. Andreani, F. Jamme, Q. P. Vanbellingen, A. Brunelle, L. Richard, P. Dumas, M. Réfrégiers, Abiotic synthesis of amino acids in the recesses of the oceanic lithosphere. *Nature* **564**, 59–63 (2018).

19. M. Andreani, B. Ménez, New perspectives on abiotic organic synthesis and processing during hydrothermal alteration of the oceanic lithosphere, in *Deep Carbon: Past to Present*, B. N. Orcutt, I. Daniel, R. Dasgupta, Eds. (Cambridge Univ. Press, 2019), pp. 447–479.

20. M. C. Sforna, D. Brunelli, C. Pisapia, V. Pasini, D. Malferrari, B. Ménez, Abiotic formation of condensed carbonaceous matter in the hydrating oceanic crust. *Nat. Commun.* **9**, 5049 (2018).

21. D. A. Sverjensky, V. Stagno, F. Huang, Important role for organic carbon in subduction-zone fluids in the deep carbon cycle. *Nat. Geosci.* **7**, 909–913 (2014).

22. R. Tao, L. Zhang, M. Tian, J. Zhu, X. Liu, J. Liu, H. E. Höfer, V. Stagno, Y. Fei, Formation of abiotic hydrocarbon from reduction of carbonate in subduction zones: Constraints from petrological observation and experimental simulation. *Geochim. Cosmochim. Acta* **239**, 390–408 (2018).

23. F. Huang, I. Daniel, H. Cardon, G. Montagnac, D. A. Sverjensky, Immiscible hydrocarbon fluids in the deep carbon cycle. *Nat. Commun.* **8**, 15798 (2017).

24. A. Vitale Brovarone, I. Martinez, A. Elmaleh, R. Compagnoni, C. Chaduteau, C. Ferraris, I. Esteve, Massive production of abiotic methane during subduction evidenced in metamorphosed ophicarbonates from the Italian Alps. *Nat. Commun.* **8**, 14134 (2017).

25. A. Vitale-Brovarone, D. A. Sverjensky, F. Piccoli, F. Ressico, D. Giovannelli, I. Daniel, Subduction hides high-pressure sources of energy that may feed the deep subsurface biosphere. *Nat. Commun.* **11**, 3880 (2020).

26. A. Boutier, A. Vitale Brovarone, I. Martinez, O. Sissmann, S. Mana, High-pressure serpentinization and abiotic methane formation in metaperidotite from the Appalachian subduction, northern Vermont. *Lithos* **396–397**, 106190 (2021).

27. J. Zhu, L. Zhang, R. Tao, Y. Fei, The formation of graphite-rich eclogite vein in S.W. Tianshan (China) and its implication for deep carbon cycling in subduction zone. *Chem. Geol.* **533**, 119430 (2020).

28. M. E. Galvez, O. Beyssac, I. Martinez, K. Benzerara, C. Chaduteau, B. Malvoisin, J. Malavieille, Graphite formation by carbonate reduction during subduction. *Nat. Geosci.* **6**, 473–477 (2013).

29. P. Bouilhol, B. Debret, E. C. Inglis, M. Warembourg, T. Grocolas, T. Rigaudier, J. Villeneuve, K. W. Burton, Decoupling of inorganic and organic carbon during slab mantle devolatilisation. *Nat. Commun.* **13**, 308 (2022).

30. M. Zolotov, E. Shock, Abiotic synthesis of polycyclic aromatic hydrocarbons on Mars. *J. Geophys. Res. E Planets.* **104**, 14033–14049 (1999).

31. A. D. Chanyshev, K. D. Litasov, A. F. Shatskiy, I. S. Sharygin, Y. Higo, E. Ohtani, Transition from melting to carbonization of naphthalene, anthracene, pyrene and coronene at high pressure. *Phys. Earth Planet. Inter.* **270**, 29–39 (2017).

32. O. Plümper, H. E. King, T. Geisler, Y. Liu, S. Pabst, I. P. Savov, D. Rost, T. Zack, Subduction zone forearc serpentinites as incubators for deep microbial life. *Proc. Natl. Acad. Sci. U.S.A.* **114**, 4324–4329 (2017).

33. P. Fryer, J. P. Lockwood, N. Becker, S. Phipps, C. S. Todd, Significance of serpentine mud volcanism in convergent margins, in *Ophiolites and Oceanic Crust; New Insights from Field Studies and the Ocean Drilling Program*, Y. Dilek, E. M. Moores, D. Elthon, A. Nicolas, Eds. (Geological Society of America, 2000), pp. 35–51.

34. P. Fryer, Serpentinite mud volcanism: Observations, processes, and implications. *Ann. Rev. Mar. Sci.* **4**, 345–373 (2012).

35. P. Fryer, C. G. Wheat, T. Williams; the Expedition 366 Scientists, Mariana convergent margin and south chamorro seamount. *Proc. Int. Ocean Discov. Progr.* **366**, 1–12 (2018).

36. B. Debret, E. Albers, B. Walter, R. Price, J. D. Barnes, H. Beunon, S. Facq, D. P. Gillikin, N. Mattielli, H. Williams, Shallow forearc mantle dynamics and geochemistry: New insights from the IODP expedition 366. *Lithos* **326–327**, 230–245 (2019).

37. B. Debret, C. D. J. Reekie, N. Mattielli, H. Beunon, B. Ménez, I. P. Savov, H. M. Williams, Redox transfer at subduction zones: Insights from Fe isotopes in the Mariana forearc. *Geochemical Perspect. Lett.* **12**, 46–51 (2020).

38. B. W. Evans, The serpentinite multisystem revisited: Chrysotile is metastable. *Int. Geol. Rev.* **46**, 479–506 (2004).

39. S. Schwartz, S. Guillot, B. Reynard, R. Lafay, B. Debret, C. Nicollet, P. Lanari, A. L. Auzende, Pressure-temperature estimates of the lizardite/antigorite transition in high pressure serpentinites. *Lithos* **178**, 197–210 (2013).

40. D. Canil, H. S. C. O'Neill, D. G. Pearson, R. L. Rudnick, W. F. McDonough, D. A. Carswell, Ferric iron in peridotites and mantle oxidation states. *Earth Planet. Sci. Lett.* **123**, 205–220 (1994).

41. V. J. M. Salters, A. Stracke, Composition of the depleted mantle. *Geochem. Geophys. Geosyst.* **5**, Q05B07 (2004).

42. A. Delacour, G. L. Früh-Green, S. M. Bernasconi, P. Schaeffer, D. S. Kelley, Carbon geochemistry of serpentinites in the Lost City Hydrothermal System (30°N, MAR). *Geochim. Cosmochim. Acta* **72**, 3681–3702 (2008).

43. E. M. Schwarzenbach, G. L. Früh-Green, S. M. Bernasconi, J. C. Alt, A. Plas, Serpentinization and carbon sequestration: A study of two ancient peridotite-hosted hydrothermal systems. *Chem. Geol.* **351**, 115–133 (2013).

44. E. M. Schwarzenbach, M. J. Caddick, M. Petroff, B. C. Gill, E. H. G. Cooperdock, J. D. Barnes, Sulphur and carbon cycling in the subduction zone mélange. *Sci. Rep.*, 15517 (2018).

45. E. Cannaò, M. Tiepolo, G. E. Bebout, M. Scambelluri, Into the deep and beyond: Carbon and nitrogen subduction recycling in secondary peridotites. *Earth Planet. Sci. Lett.* **543**, 116328 (2020).

46. K. Kashefi, D. R. Lovley, Extending the upper temperature limit for life. *Science* **301**, 934 (2003).

47. A. Y. Lein, Authigenic carbonate formation in the ocean. *Lithol. Miner. Resour.* **39**, 1–30 (2004).

48. D. L. Valentine, W. S. Reeburgh, New perspectives on anaerobic methane oxidation. *Envir. Microbiol.* **2**, 477–484 (2000).

49. E. Frery, P. Fryer, W. Kurz, A. Nguyen, O. Sissmann, T. Uysal, J. Zhao, Episodicity of structural flow in an active subduction system, new insights from mud volcano's carbonate veins – Scientific Ocean drilling expedition IODP 366. *Mar. Geol.* **434**, 106431 (2021).

50. M. Freitas, V. H. Magalhães, M. R. Azevedo, L. Pinheiro, E. Salgueiro, F. F. Abrantes, Authigenic carbonate precipitation at the Yinazao serpentinite mud volcano. *Comun. Geol.* **107**, 61–64 (2019).

51. J. A. Haggerty, Petrology and geochemistry of neogene sedimentary rocks from Mariana Forearc seamounts: Implications for emplacement of the seamounts, in *Seamounts Islands and Atolls, Geophysical Monograph Series 43*, B. Keating, P. Fryer, R. Batiza, G. Boehlert, Eds. (AGU, 1987), pp. 175–185.

52. R. Popa, B. K. Kinkle, A. Badescu, Pyrite framboids as biomarkers for iron-sulfur systems. *Geomicrobiol. J.* **21**, 193–206 (2004).

53. G. E. Bebout, M. L. Fogel, P. Cartigny, Nitrogen: Highly volatile yet surprisingly compatible. *Elements* **9**, 333–338 (2013).

54. B. Debret, P. Bouilhol, M. L. Pons, H. Williams, Carbonate transfer during the onset of slab devolatilization: New insights from Fe and Zn stable isotopes. *J. Petrol.* **59**, 1145–1166 (2018).

55. I. P. Savov, J. G. Ryan, M. D'Antonio, P. Fryer, Shallow slab fluid release across and along the Mariana arc-basin system: Insights from geochemistry of serpentinized peridotites from the Mariana fore arc. *J. Geophys. Res. Solid Earth* **112**, B09205 (2007).

56. B. Debret, H. Beunon, N. Mattielli, M. Andreani, I. Ribeiro da Costa, J. Escartin, Ore component mobility, transport and mineralization at mid-oceanic ridges: A stable isotopes (Zn, Cu and Fe) study of the Rainbow massif (Mid-Atlantic Ridge 36°14′N). *Earth Planet. Sci. Lett.* **503**, 170–180 (2018).

57. S. J. Sadofsky, G. E. Bebout, Nitrogen geochemistry of subducting sediments: New results from the Izu-Bonin-Mariana margin and insights regarding global nitrogen subduction. *Geochem. Geophys. Geosyst.* **5**, Q03I15 (2004).

58. D. R. Hilton, T. P. Fischer, E. Hauri, A. M. Shaw, Controls on the He–C systematics of the Izu-Bonin-Marianas (IBM) subduction zone. *Geochim. Cosmochim. Acta* **70**, A252 (2006).

59. J. A. Resing, E. T. Baker, J. E. Lupton, S. L. Walker, D. A. Butterfield, G. J. Massoth, K.-I. Nakamura, Chemistry of hydrothermal plumes above submarine volcanoes of the mariana arc. *Geochem. Geophys. Geosyst.* **10**, Q02009 (2009).

60. N. Bellot, M. Boyet, R. Doucelance, P. Bonnand, I. P. Savov, T. Plank, T. Elliott, Origin of negative cerium anomalies in subduction-related volcanic samples: Constraints from Ce and Nd isotopes. *Chem. Geol.* **500**, 46–63 (2018).

61. C. G. Macpherson, D. R. Hilton, K. Hammerschmidt, No slab-derived CO_2 in Mariana Trough back-arc basalts: Implications for carbon subduction and for temporary storage of CO_2 beneath slow spreading ridges. *Geochem. Geophys. Geosyst.* **11**, Q11007 (2010).

62. C. D. Menzies, R. E. Price, J. Ryan, O. Sissmann, K. Takai, C. G. Wheat, Spatial variation of subduction zone fluids during progressive subduction: Insights from Serpentinite mud volcanoes. *Geochim. Cosmochim. Acta* **319**, 118–134 (2022).

63. P. Eickenbusch, K. Takai, O. Sissman, S. Suzuki, C. Menzies, S. Sakai, P. Sansjofre, E. Tasumi, S. M. Bernasconi, C. Glombitza, B. B. Jørgensen, Y. Morono, M. A. Lever, Origin of short-chain organic acids in serpentinite mud volcanoes of the Mariana convergent margin. *Front. Microbiol.* **10**, 1–21 (2019).

64. C. Marche, C. Ferronato, J. Jose, Solubilities of *n*-Alkanes (C_6 to C_8) in water from 30 °C to 180 °C. *J. Chem. Eng. Data* **48**, 967–971 (2003).

65. C. Cai, D. A. Wiens, W. Shen, M. Eimer, Water input into the Mariana subduction zone estimated from ocean-bottom seismic data. *Nature* **563**, 389–392 (2018).

66. V. Magni, P. Bouilhol, J. van Hunen, Deep water recycling through time. *Geochem. Geophys. Geosyst.* **15**, 4203–4216 (2014).

67. J. M. Ribeiro, C. T. A. Lee, An imbalance in the deep water cycle at subduction zones: The potential importance of the fore-arc mantle. *Earth Planet. Sci. Lett.* **479**, 298–309 (2017).

68. P. D. Clift, A revised budget for Cenozoic sedimentary carbon subduction. *Rev. Geophys.* **55**, 97–125 (2017).

69. T. Nagaya, A. M. Walker, J. Wookey, S. R. Wallis, K. Ishii, J.-M. Kendall, Seismic evidence for flow in the hydrated mantle wedge of the Ryukyu subduction zone. *Sci. Rep.* **6**, 29981 (2016).

70. R. Lin, G. P. Ritz, Studying individual macerals using i.r. microspectrometry, and implications on oil versus gas/condensate proneness and "low-rank" generation. *Org. Geochem.* **20**, 695–706 (1993).

71. V. Pasini, D. Brunelli, P. Dumas, C. Sandt, J. Frederick, K. Benzerara, S. Bernard, B. Ménez, Low temperature hydrothermal oil and associated biological precursors in serpentinites from mid-ocean ridge. *Lithos* **178**, 84–95 (2013).

72. B. H. Stuart, *Infrared Spectroscopy: Fundamentals and Applications* (John Wiley & Sons, 2004).

73. D. Bonnemains, J. Carlut, J. Escartín, C. Mével, M. Andreani, B. Debret, Magnetic signatures of serpentinization at ophiolite complexes. *Geochem. Geophys. Geosyst.* **17**, 2969–2986 (2016).

74. L. B. Stokking, D. L. Merrill, R. B. Haston, J. R. Ali, K. L. Saboda, Rock magnetic studies of serpentinite seamounts in the Mariana and Izu-Bonin regions, in *Proceedings of the Ocean Drilling Program, 125 Scientific Results*, P. Fryer, J. A. Pearce, L. B. Stokking, Eds. (College Station, TX, 1992), pp. 561–579; doi:10.2973/odp.proc.sr.125.158.1992.

75. Shipboard Scientific Party, SITE 1200, in *Proceedings of the Ocean Drilling Program, Initial Reports*, M. H. Salisbury, M. Shinohara, C. Richter, Eds. (College Station, TX, 2002), vol. 195, pp. 1–173; http://www-odp.tamu.edu/publications/195_IR/chap_03/chap_03.htm.

76. A. P. Roberts, L. Chang, C. J. Rowan, C.-S. Horng, F. Florindo, Magnetic properties of sedimentary greigite (Fe3S4): An update. *Rev. Geophys.* **49**, RG1002 (2011).
